# Dynamics of culture-recovered cotton root-associated fungi under charcoal rot disease and preventive management regimes

**DOI:** 10.3389/ffunb.2026.1928692

**Published:** 2026-10-12

**Authors:** Eden Etedgi, Daniel Demenchuk, Elhanan Dimant, Peleg Hadari, Ofir Degani

**Affiliations:** 1 Faculty of Sciences, Tel-Hai University of Kiryat Shmona in the Galilee, Kiryat Shmona, Israel; 2 Migal – Galilee Research Institute, Kiryat Shmona, Israel

**Keywords:** 6-pentyl-α-pyrone, azoxystrobin, crop protection, fungal metabolites, integrated disease management, *Macrophomina phaseolina*, real-time PCR, Trichoderma

## Abstract

Charcoal rot (CRD), caused by *Macrophomina phaseolina*, is a major soil-borne disease of cotton, and conventional chemical control often provides inconsistent suppression. This study aimed to characterize management-associated patterns among culture-recovered cotton root-associated fungi and to identify fungal interactions relevant to *M. phaseolina* suppression. Roots were collected from asymptomatic and symptomatic cotton plants at mid-season and end-season in a field subjected to chemical, *Trichoderma*-based, and integrated CRD management. Root-associated isolates were recovered by culture-dependent methods and identified using molecular markers and phylogenetic analyses. Selected fungal taxa were evaluated *in vitro* and in a 33-day greenhouse seedling assay, while commercial 6-pentyl-α-pyrone (6-PP) was examined in a targeted antifungal assay. From approximately 300 recovered isolates, 66 representative morphotypes were selected and molecularly identified, including 56 fungal and 10 bacterial isolates. *Aspergillus* was the most frequently recovered fungal genus at mid-season, whereas *Alternaria* became predominant at season’s end, particularly in symptomatic plants. The numbers of recovered fungal isolates and taxa increased modestly in asymptomatic plants but more strongly in symptomatic plants, while *Trichoderma*-based management was associated with markedly lower numbers of recovered fungal isolates and taxa. Commercial 6-PP inhibited *M. phaseolina* in a concentration- and time-dependent manner (94.5–100% at nominal 400–800 µg mL^-^¹ through 72 h). Greenhouse assays showed limited effects on early seedling development during the latent disease stage. Nevertheless, qPCR revealed mean reductions of 92%, 94%, and 99% in M. phaseolina DNA following co-inoculation with A. niger, N. solani, and A. alternata, respectively, compared with 47% for *R. arrhizus*. Only the reduction associated with *A. alternata* remained significant after multiplicity adjustment. These findings associate biological and integrated management with distinct patterns in culture-recovered root-associated fungi and identify microbial interactions and natural product activity relevant to future CRD management.

## Graphical Abstract

**Figure:**
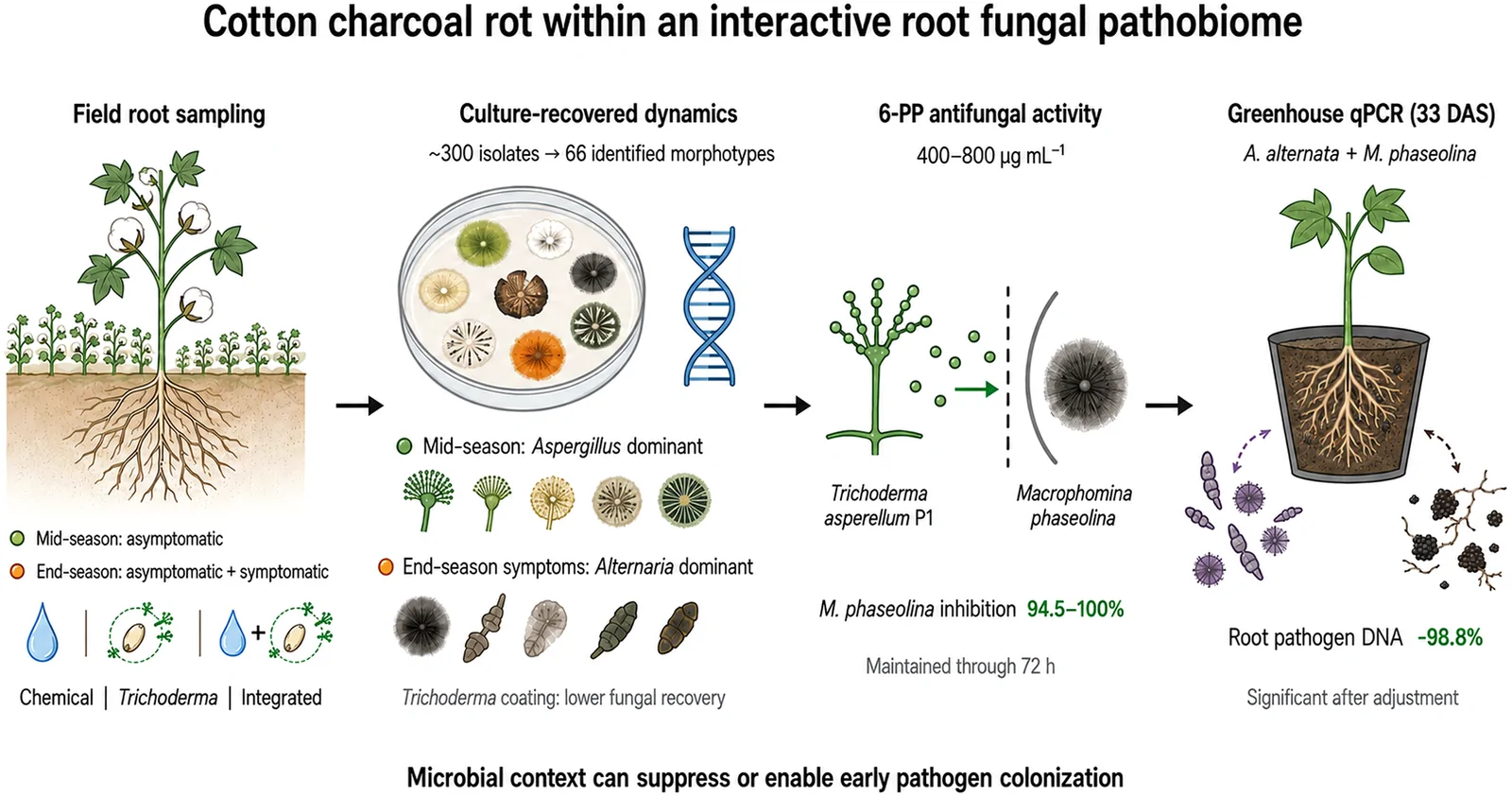

## 1 Introduction

Cotton (*Gossypium* spp.) is the world’s most important natural fiber crop and a critical source of income for millions of farmers. In 2023, cotton was cultivated on roughly 32 million hectares (FAOSTAT, 2023), producing more than 24 million metric tons of lint (U.S. Department of Agriculture, Foreign Agricultural Service, Production – Cotton 2025). Recent assessments (Ashraf et al., 2024; Saleem et al., 2024) estimate that cotton generates an annual global economic impact of approximately 600 billion USD, making it the leading natural fiber in both production and trade. The cotton sector supports nearly one billion people worldwide, including approximately 250 million individuals directly employed or economically dependent on cotton-related activities. Cotton is cultivated, processed, or traded in about 150 countries and directly sustains the livelihoods of roughly 100 million families. Cotton is grown across temperate, subtropical, and tropical regions, but its future productivity is increasingly threatened by climate change (Bange et al., 2016), which is expected to intensify the prevalence and severity of soil-borne diseases, such as cotton charcoal rot disease (CRD) (Cohen et al., 2022). Biotic stresses, especially fungal pathogens and insect pests, significantly reduce yields and contribute to rising production costs due to the need for extensive control measures.

Among these threats, *Macrophomina phaseolina* (Tassi) Goid., the causal agent of CRD, is one of the most destructive. This pathogen has an exceptionally broad host range, infecting more than 500 plant species across roughly 75 botanical families (Banaras et al., 2021; Marquez et al., 2021). Disease severity in cotton is influenced by soil infestation levels, pathogen virulence variability, and the host’s inherent susceptibility (Su et al., 2001). Symptoms typically appear during flowering and boll development and include wilting, stem and leaf desiccation, and ultimately, plant death (Degani et al., 2020). Yield losses can be substantial and highly localized, with complete plant mortality often observed in affected field patches (Yadav et al., 2021).

The persistence of *M. phaseolina* in soil and plant residues is largely attributed to its production of melanized microsclerotia and pycnidia (Marquez et al., 2021)—robust, multicellular propagules that confer long-term survival and significantly complicate disease management (Lodha and Mawar, 2020; Kumar et al., 2023; Alizadeh et al., 2025). Existing control strategies include crop rotation, the use of less susceptible cultivars, cultural practices, soil solarization, and optimized irrigation regimes to reduce disease pressure (Lodha and Mawar, 2020). However, these approaches typically require extended implementation periods and are often challenged by the pathogen’s strong saprophytic capacity, which allows it to thrive on plant residues and persist in the soil (Ghosh et al., 2018; Kumar et al., 2023). Thus, given the lack of highly resistant cotton cultivars, systemic fungicides remain the primary means of suppressing CRD (Bashir et al., 2017).

Mounting concerns over the intensive use of synthetic fungicides, along with the emergence of fungicide-resistant pathogen strains (Shcherbakova, 2019), underscore the urgent need for alternative, environmentally sustainable CRD management solutions (Banaras et al., 2021; Javed et al., 2021; Uroos et al., 2022). The deployment of agriculturally beneficial microorganisms offers a promising ecological approach, providing effective disease suppression while mitigating risks to ecosystems and human health (Degani, 2025). Notably, plant associations with *Trichoderma* spp. commonly result in enhanced growth and reduced disease incidence compared with untreated plants (Nakkeeran et al., 2016; Elshahawy and El-Sayed, 2018; De Oliveira et al., 2021; Degani and Dor, 2021; Gordani et al., 2023). Several *Trichoderma* species have shown strong antagonistic activity against *M. phaseolina* across diverse experimental platforms, including *in vitro* dual-culture assays, controlled seedling trials, and full-scale commercial field evaluations (Iqbal and Mukhtar, 2020; Degani et al., 2023a, 2024b; Waheed et al., 2024).

While these studies aimed to develop proof-of-concept sustainable solutions for CRD, their effects on the plant root mycobiome, including beneficial endophytes and the broader pathobiome, have been insufficiently explored. This knowledge gap limits our ability to manipulate and reinforce beneficial members of the plant-associated, particularly root-associated microbiome, to enhance host immunity. Moreover, some root-associated colonists are latent opportunists or active pathogens that may become disease-causing when microbial equilibrium is disrupted, for example, following the suppression of conventional chemical or biological control interventions (Degani et al., 2025).

The current study aimed to characterize shifts in the culture-recovered root-associated fungi under commercial field conditions and different protective-management regimes with azoxystrobin (applied by sprinkling or irrigation) and *Trichoderma* spp. (applied as a seed coating), used either individually or in combination. The agronomic performance of these treatments was recently evaluated (Degani et al., 2024b), demonstrating the strong potential of biological and integrated biological–chemical management approaches to mitigate CRD under high disease pressure. In the field trial, biological and integrated treatments increased cotton yield by up to 17%, reduced visible disease symptoms by up to 27%, and decreased *M. phaseolina* DNA levels in roots by up to 37%, performing similarly to or better than standard chemical management. When treatments were grouped by management strategy, the integrated biological–chemical approach conferred a significant advantage in plant health (*p* < 0.05) over purely chemical interventions.

During the same growing season, cotton roots were collected for the present analysis of culture-recovered fungal taxon richness. Isolates were grouped by colony morphology and identified using molecular markers, and representative isolates from dominant fungal genera were evaluated for direct, diffusible, and volatile interactions with *M. phaseolina*. Because *Trichoderma asperellum* (strain P1) was a component of the biological seed-coating treatment and had previously demonstrated strong antifungal activity, its antagonistic effects were examined across complementary *in vitro* assay formats. To relate *T. asperellum* activity to a chemically defined natural product, commercial 6-PP, previously isolated and identified from *T. asperellum* culture medium (Degani et al., 2021), was tested against *M. phaseolina*, with *M. maydis* included as a previously characterized reference fungus. Finally, a greenhouse seedling assay conducted during the CRD-sensitive pathogen invasion stage evaluated the biological relevance of selected fungal interactions during early pathogen establishment.

## 2 Materials and methods

### 2.1 Rationale and research design

This study investigated the culture-recovered fungi under field conditions characterized by high CRD pressure and chemical, biological, or integrated management interventions. The field component was based on root sampling from a previously established commercial field trial (Degani et al., 2024b), rather than a replicated microbiome-manipulation experiment; therefore, these data are interpreted as observational evidence of associations among management regime, visual symptom status, sampling time, and the recovered root-associated fungi. To strengthen biological interpretation, representative field-derived taxa were subsequently tested under controlled *in vitro* and greenhouse conditions for functional interactions with *M. phaseolina*. Thus, the study combines field-derived observational sampling with controlled functional testing, enabling identification of management-associated microbial patterns and candidate antagonistic interactions without inferring direct causality for all field-level patterns in the culture-recovered fungal collection.

No pretreatment soil or root samples were collected. Because treatment application began at or immediately after sowing via biological seed coating and chemical applications, root tissues were not available before treatment exposure. In addition, bulk soil would not provide a directly comparable baseline for the culture-recovered root endosphere-associated fungi examined here. Early seedling roots would already represent a post-treatment, developmentally distinct sample rather than a true pretreatment baseline. Accordingly, management regimes were compared within each sampling time, and differences between sampling dates were interpreted as developmental-stage-associated temporal patterns.

A culture-dependent isolation approach was used to recover viable microorganisms and enable downstream analyses of individual isolates and their interactions, which are not accessible through amplicon-based profiling alone. The isolation procedure targeted endosphere-associated microorganisms, which were recovered after surface sterilization to reduce epiphytic contamination. Representative isolates were selected based on recurrence across treatments and/or association with pathogenic or opportunistic taxa. This workflow links field sampling with laboratory interaction assays and greenhouse functional testing, providing a practical approach for identifying root-associated microorganisms and candidate microbial interactions that may support future CRD management.

### 2.2 *Macrophomina phaseolina* strain origin and growth of the fungi

The phytopathogenic fungus *M. phaseolina* (isolate Mp-1) was isolated in 2017 from diseased cotton plants at the laboratory of Roni Cohen, Newe Ya’ar Research Center, Israel. Species identification was confirmed based on physiological characterization, colony morphology, microscopic features, and molecular analyses (Degani et al., 2020, 2023a). The pathogenicity of this isolate toward cotton has been demonstrated in multiple previous studies (Degani et al., 2022, 2023b, 2024b). The fungus was maintained on potato dextrose agar (PDA; Sisco Research Laboratories, Detroit, MI) in 90-mm Petri dishes incubated at 28 ± 1 °C in darkness for 4–5 days using MaxQ™ 6000 Incubated/Refrigerated Stackable Shakers (Thermo Fisher Scientific, Waltham, MA). Subculturing was performed by transferring a 6-mm-diameter mycelial plug excised from the edge of a colony onto a fresh PDA plate, followed by incubation under identical conditions.

### 2.3 Isolation and identification of culturable cotton root-associated microorganisms

At each sampling date, one cotton plant was randomly selected from each of the 84 field plots, representing different CRD management treatments (Degani et al., 2024b), yielding 84 independent plot-level root samples per sampling date and 168 samples in total. The plots were equally distributed among the chemical, biological, and integrated management regimes (*n* = 28 plots per regime). At mid-season (74 days after sowing), all sampled plants lacked visible CRD symptoms, with 28 samples collected per management regime. At the end of the season (151 days after sowing), 28 plants were sampled from each regime, equally divided between plants without visible symptoms and those displaying typical CRD symptoms (*n* = 14 per category per regime; 42 per category overall). Because molecular testing was not performed prior to isolation, latent *M. phaseolina* infection could not be excluded in the asymptomatic group. Samples from different field plots were processed separately and were not pooled; therefore, each field plot represented an independent field-level sampling unit. From each selected plant, two to three root segments (10–15 cm in length) were collected as subsamples of the corresponding plot-level root sample and used for the isolation.

Endophytic microorganisms were isolated from surface-disinfected internal cotton root tissues following a previously published protocol (Degani et al., 2024a) with minor modifications. Before isolation, the roots were rinsed under running tap water to remove adhering soil and debris, immersed in 1% sodium hypochlorite for 1 min, treated twice with 70% ethanol, and rinsed twice with sterile double-distilled water. The outer peel was then removed aseptically, and transverse sections were excised from the innermost tissues using a sterile scalpel and placed on 90-mm PDA plates. Only internal tissues were plated to minimize the recovery of microorganisms remaining on the root surface. The effectiveness of surface disinfection was not independently verified.

The plates were incubated at 28 ± 1 °C in darkness for 2–3 days. Emerging fungal and bacterial colonies were subcultured onto fresh PDA plates by excising small agar plugs from the actively growing margins of the colonies. The new plates were incubated under identical conditions for an additional 5–6 days, during which isolate growth characteristics were monitored. This process was repeated until axenic pure cultures were obtained. Approximately 300 isolates were recovered and grouped based on macroscopic colony characteristics. From this collection, 66 representative morphotypes were selected and molecularly identified, comprising 56 fungal and 10 bacterial isolates (**Table 1**). Selected fungal isolates were subsequently evaluated in interaction assays. This morphotype-selected collection represents only a subset of the PDA-recoverable root-associated microorganisms and cannot define the composition or richness of the complete root microbial community.

**Table 1:** Summary of culturable cotton root-associated bacterial and fungal isolates.

Parameter	Treatment	Mid-season	End-season		
		Asymptomatic	Asymptomatic	Symptomatic	Total
Root samples		84	42	42	**168**
Selected representative isolates		14	18	34	**66**
Number of bacterial isolates		**3**	**5**	**2**	**10**
Bacterial species richness		3	5	2	**8**
Bacterial genus richness		2	4	2	**5**
Number of fungal isolates	Undefined	2	–	1	**3**
	Chemical treatment	2	4	10	**16**
	Biological treatment	4	1	8	**13**
	Integrated treatment	3	8	13	**24**
Total fungal isolates		**11**	**13**	**32**	**56**
Fungal species richness	Chemical treatment	2	3	7	**12**
	Biological treatment	4	1	4	**7**
	Integrated treatment	3	7	11	**15**
Total fungal species		**7**	**10**	**16**	**24**
Fungal genus richness	Chemical treatment	2	3	5	**8**
	Biological treatment	3	1	4	**5**
	Integrated treatment	3	5	6	**8**
Total fungal genera		**5**	**7**	**10**	**11**

For descriptive comparisons, the recovered fungi were grouped by genus (**Figure 5**) or management regime (**Figure 6**). Taxon richness was defined as the number of distinct fungal taxa assigned to each genus or recovered under each management regime, whereas isolate richness was defined as the corresponding total number of recovered isolates. Within each sampling and symptom category, relative proportions were calculated by dividing each group-specific taxon or isolate count by the corresponding total and multiplying by 100. For **Figure 6**, taxa recovered under more than one management regime were counted once within each applicable regime, and isolates without a defined management assignment were excluded. Bold values indicate totals. Pooled species and genus richness count distinct taxa and are not necessarily additive across categories.

### 2.4 Molecular identification of endophytes

Genomic DNA was extracted from PDA-grown endophytic mycelia using the MasterPure™ Yeast DNA Purification Kit (Sigma, Rehovot, Israel), according to the manufacturer’s instructions. Molecular identification was performed by PCR amplification and sequencing of ribosomal DNA regions using the universal ITS4 and ITS5 primers (**Table 2**), targeting the fungal internal transcribed spacer (ITS) region. For bacterial isolates, the V3 region of the 16S rRNA gene was amplified and sequenced using the 338F/806R primers. PCR reactions were performed in a total volume of 20 µL using a Rapidcycler system (Idaho Technology, Salt Lake City, UT). They contained 1 µL of each primer (20 µM), 10 µL of RedTaq^®^ ReadyMix (Sigma), 3 µL of template DNA, and 5 µL of autoclaved DDW. Thermal cycling conditions consisted of an initial denaturation at 94 °C for 2 min, followed by 30 cycles of 94 °C for 30 s, 55 °C for 30 s, and 72 °C for 1 min, with a final extension at 72 °C for 5 min (Degani et al., 2024a). Amplified products were stored at 4 °C until sequencing. Sequencing was carried out bidirectionally using ITS4 and ITS5 or the 338F and 806R primers by Hy Labs (Rehovot, Israel). The resulting sequences were compared against the GenBank database using the BLASTN algorithm (NCBI, Bethesda, MD; accessed 15 January 2026). *Fusariu*m isolates were further validated using pairwise alignment against the Fusarioid-ID database (www.fusarium.org; accessed 15 January 2026). Newly generated fungal ITS and bacterial 16S rRNA gene sequences were deposited in GenBank. Closest BLASTN matches, sequence identity values, and isolate metadata are provided in **Supplementary Table S2**, and accession numbers are listed in **Supplementary Table S3**.

**Table 2:** Primers used in this study.

Pairs	Primer	Sequence	Uses	Amplification	References
Pair 1	MpKFI MpKRI	5′-CCGCCAGAGGACTATCAAAC-3′ 5′-CGTCCGAAGCGAGGTGTATT-3′	qPCR Target gene	*M. phaseolina* species-specific fragment (300–400 bp)	(Babu et al., 2007)
Pair 2	COX-F COX-R	5′-GTATGCCACGTCGCATTCCAGA-3′ 5′-CAACTACGGATATATAAGRRCCRRAACTG-3′	Plant DNA internal control	Cytochrome c oxidase (COX) gene product (~250–400 bp)	(Weller et al., 2000; Li et al., 2006)
Pair 3	ITS4 ITS5	5′-TCCTCCGCTTATTGATATGC-3′ 5′-GGAAGTAAAAGTCGTAACAAGG-3′	Fungal ITS amplification	Internal transcribed spacer region of nuclear ribosomal DNA (560 bp)	(White et al., 1990)
Pair 4	338F 806R	5′-ACTCCTACGGGAGGCAGCAG-3′ 5′-TGCTGCCTCCCGTAGGAGT-3′	Universal bacterial target gene	V3 region of the bacterial 16S rRNA gene (~170–200 bp)	(Mao et al., 2012)

### 2.5 Phylogenetic analysis

ITS sequence alignment and phylogenetic analyses were conducted using SeaView software version 5.0 (http://doua.prabi.fr/software/seaview; accessed on 15 January 2026) (Gouy et al., 2010). Multiple sequence alignment was performed with the CLUSTALW algorithm (https://www.genome.jp/tools-bin/clustalw; accessed on 15 January 2026). Phylogenetic trees were reconstructed at the DNA level using either the PhyML maximum-likelihood algorithm (Guindon et al., 2010), with branch support assessed by bootstrap analysis, or the maximum parsimony algorithm (dnapars, PHYLIP, Phylogeny Inference Package), which infers evolutionary relationships by minimizing the total number of nucleotide substitutions across all sites. The specific algorithm used is indicated for each figure. Default settings were applied, with alignment positions containing gaps excluded from the analysis. Branch support was evaluated using bootstrap resampling; **Figure 1** used 1000 replicates, whereas **Figures 2**–**4** used 100 replicates, as indicated in the figure legends. To facilitate detailed and legible presentation, the overview ITS4 tree was divided into three groups primarily according to phylogenetic proximity, with additional consideration of tree size when incorporating reference and type-strain sequences. These groups were subsequently analyzed separately in **Figures 2**–**4**.

**Figure 1:**
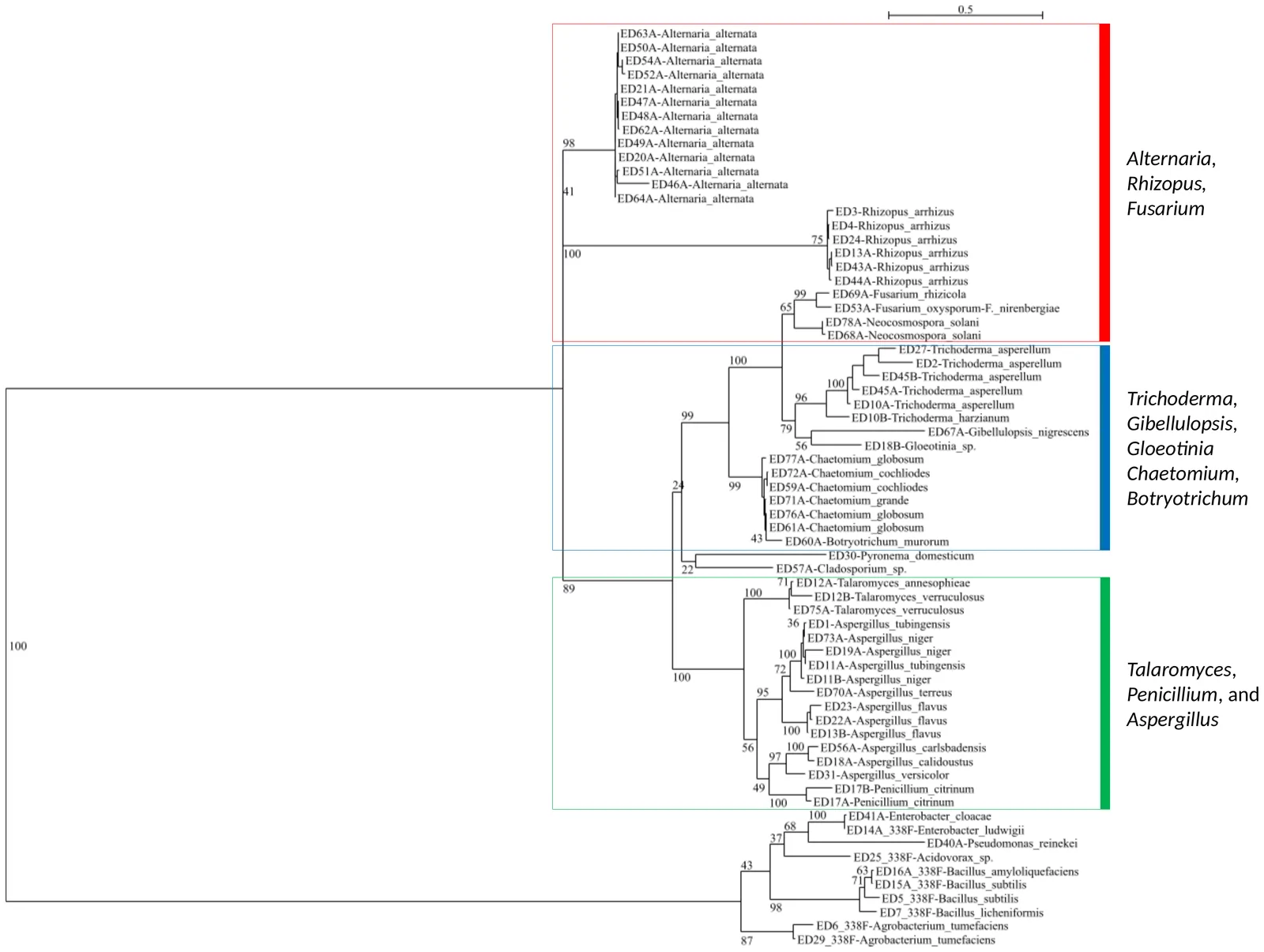
Phylogenetic analysis of the ITS4 region of fungal root isolates (listed in **Supplementary Table S2**). Sequence alignment and phylogenetic reconstruction were performed using SeaView software version 5.05 (http://doua.prabi.fr/software/seaview; accessed 10 January 2026). The phylogenetic tree was inferred using the PhyML maximum-likelihood algorithm (Guindon et al., 2010) under default parameters, with 1000 bootstrap replications to assess branch support. Bootstrap values are shown at major branches. The scale bar represents 0.5 nucleotide substitutions per site. The three groups selected for detailed phylogenetic analysis in **Figures 2**–**4** are marked in the overview tree. The groups were defined primarily by phylogenetic proximity and secondarily by practical considerations of tree size and readability. *Pyronema* and *Cladosporium*, which had limited representation among the recovered isolates, were retained only in the overview tree. Detailed clade-specific phylogenetic analyses incorporating additional reference and type strains retrieved from GenBank to support taxonomic assignment are presented in **Figures 2**–**4**.

**Figure 2:**
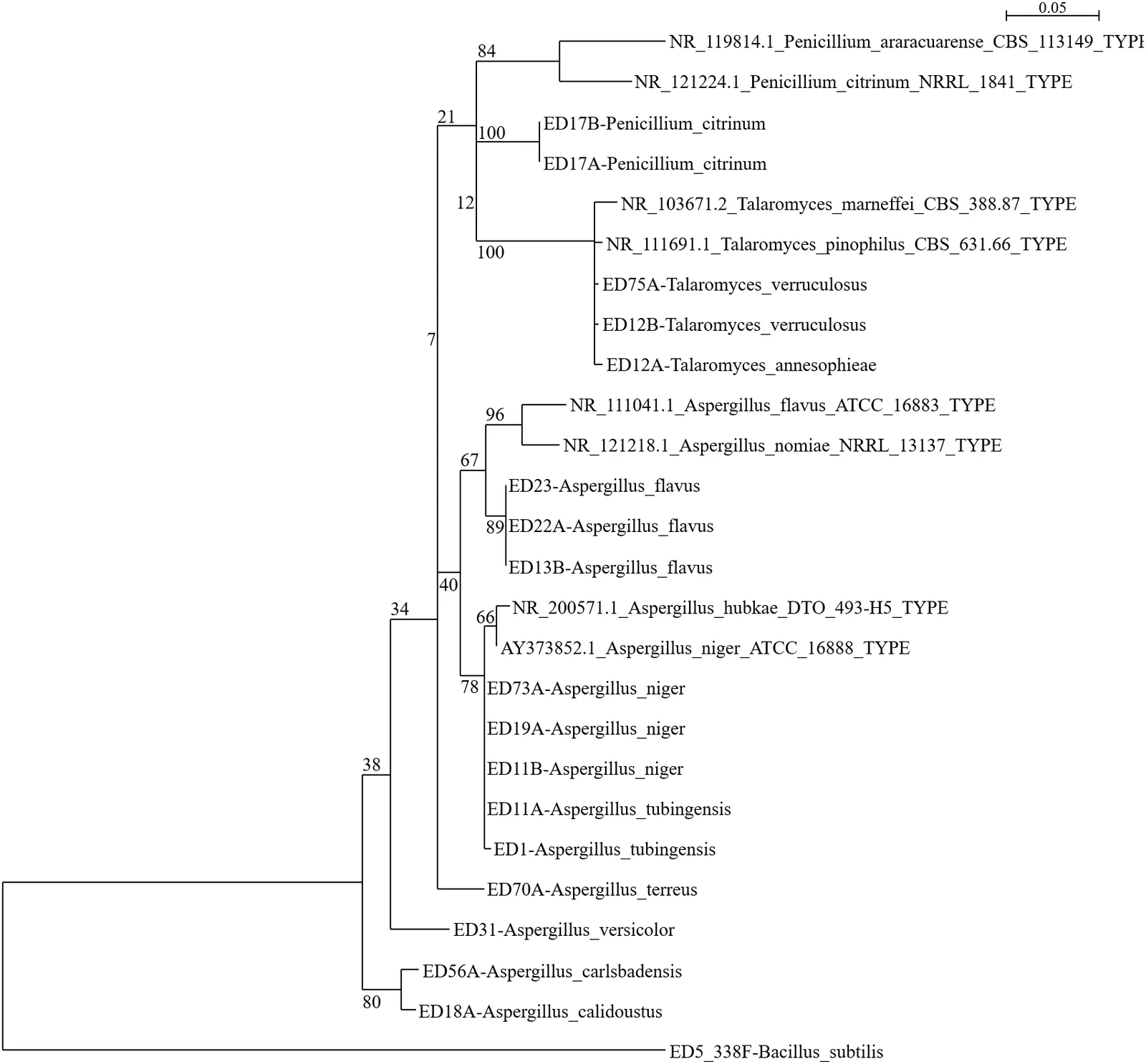
Phylogenetic analysis of the *Talaromyces*, *Penicillium*, and *Aspergillus* clade based on ITS4 sequences of cotton root endophyte isolates. The phylogenetic tree was constructed in SeaView v5.05 using the parsimony DNA-level algorithm (dnapars) with default parameters and 100 bootstrap replications. A consensus tree was generated from all calculated trees. The tree is presented as an unrooted clustering tree and was used to assess the placement of isolates relative to reference and type strains. ITS sequences from type strains (**Supplementary Table S1**), serving as official ICN nomenclatural anchors for each genus and species, were included to support taxonomic identification. The scale bar represents 5% sequence divergence. Bootstrap support values are indicated at the major branches.

**Figure 3:**
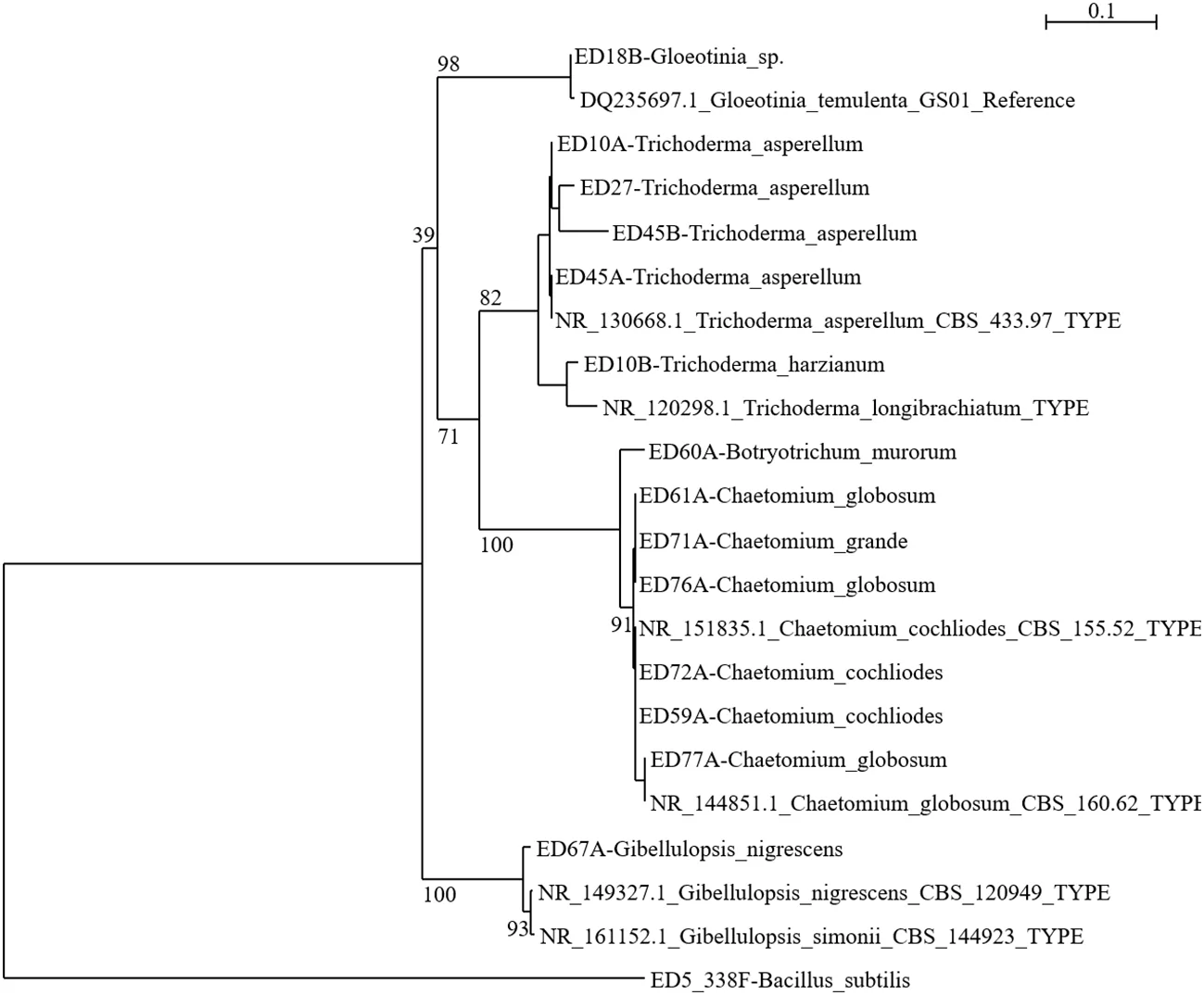
Phylogenetic analysis of the *Chaetomium*, *Botryotrichum*, *Trichoderma*, *Gibellulopsis*, and *Gloeotinia* clade based on ITS4 sequences of cotton root endophyte isolates. The phylogenetic tree was constructed in SeaView v5.05 using the parsimony DNA-level algorithm (dnapars) with default parameters and 100 bootstrap replications. A consensus tree was generated from all calculated trees. The tree is presented as an unrooted clustering tree and was used to assess the placement of isolates relative to reference and type strains. ITS sequences from type strains (**Supplementary Table S1**), serving as official ICN nomenclatural anchors for each genus and species, were included to support taxonomic identification. When type strains were unavailable, representative reference strains from well-established taxa commonly used in recent fungal taxonomic and systematic studies were employed. The scale bar represents 10% sequence divergence. Bootstrap support values are indicated at the major branches.

**Figure 4:**
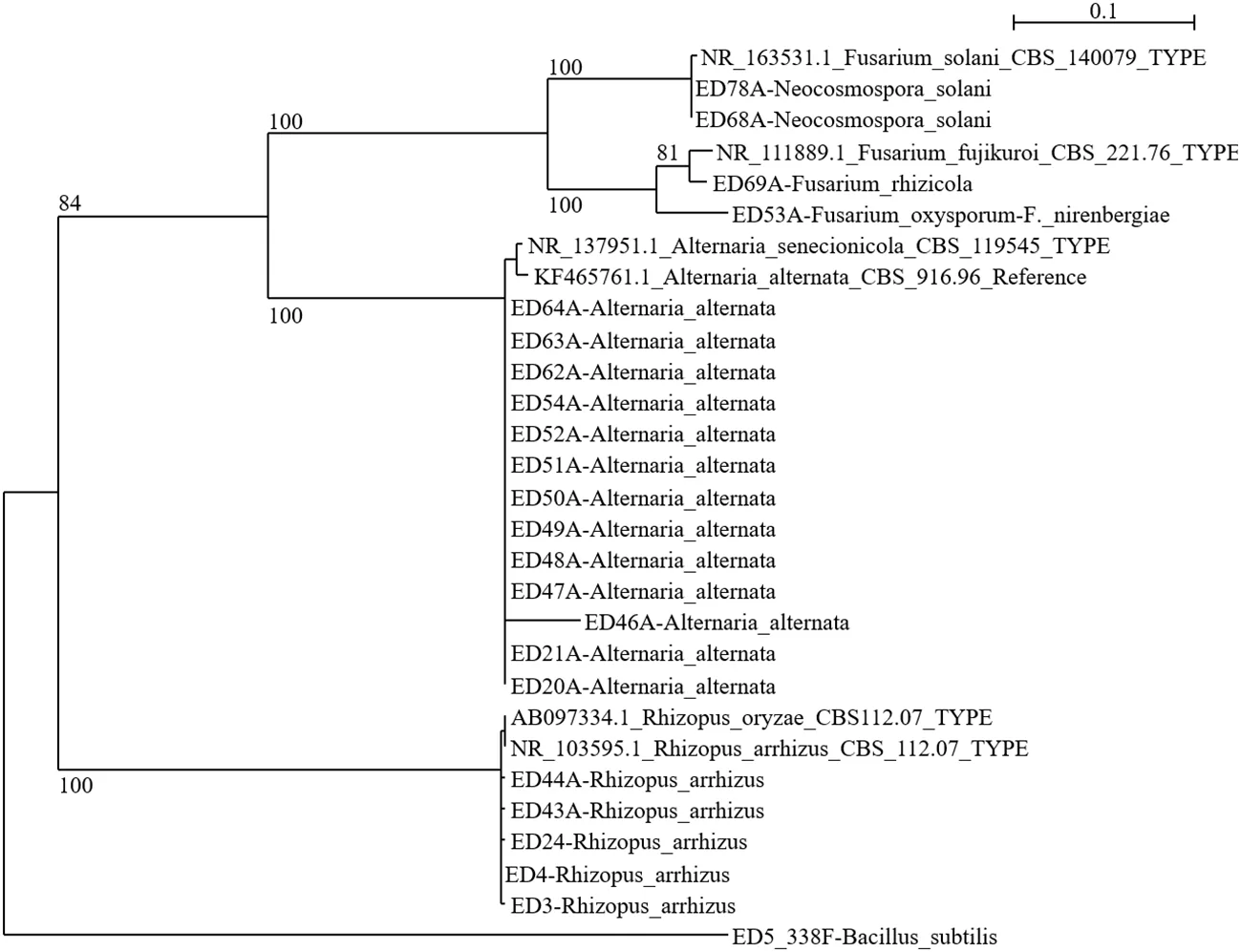
Phylogenetic analysis of the *Fusarium*, *Alternaria*, and *Rhizopus* clade based on ITS4 sequences of cotton root endophyte isolates. The phylogenetic tree was constructed in SeaView v5.05 using the parsimony DNA-level algorithm (dnapars) with default parameters and 100 bootstrap replications. A consensus tree was generated from all calculated trees. The tree is presented as an unrooted clustering tree and was used to assess the placement of isolates relative to reference and type strains. ITS sequences from type strains (**Supplementary Table S1**), serving as official ICN nomenclatural anchors for each genus and species, were included to support taxonomic identification. The scale bar represents 10% sequence divergence. Bootstrap support values are indicated at the major branches.

The trees were presented as unrooted clustering trees and were used to assess the placement of fungal isolates relative to reference and type strains. ITS sequences from type strains (**Supplementary Table S1**), which serve as official nomenclatural anchors for each genus and species according to the International Code of Nomenclature for algae, fungi, and plants (ICN), were included to support taxonomic identification. When type strains were unavailable, representative reference strains were used. In cases where BLASTN searches yielded conflicting or low-confidence species assignments between ITS4 and ITS5 sequences, final taxonomic assignment was resolved based on phylogenetic placement relative to ICN-recognized type or reference strains. Isolates that did not cluster robustly at the species level were assigned at the genus level.

### 2.6 Plate confrontation assay

Four field-derived root-associated fungal isolates were selected for the *in vitro* interaction assays: *Aspergillus niger* Ed11B, *Alternaria alternata* Ed20A, *Rhizopus arrhizus* Ed43A, and *Neocosmospora solani* Ed68A. Selection was based on taxonomic representation and ecological relevance within the culture-recovered collection. *A. niger* and *A. alternata* represented genera frequently recovered at mid-season and end-season, respectively; *R. arrhizus* represented a rapidly growing Mucorales fungus; and *N. solani* represented a *Fusarium*-like root-associated pathogen. *T. asperellum* P1 was included separately as the reference biocontrol strain used in the field seed-coating formulation. The isolates were tested against one another and against *M. phaseolina* using a previously described direct dual-culture confrontation assay (Degani et al., 2024a). Antagonistic activity was evaluated based on the secretion of antifungal compounds, inhibition of hyphal growth upon direct contact, or overgrowth of the opposing fungal colony.

For the assay, 6-mm agar disks excised from the margins of actively growing cultures were placed opposite each other, 0.5 cm from the edge of 90-mm PDA plates. Plates were incubated at 28 ± 1 °C in darkness and monitored for 4–6 days, depending on fungal growth rates. Control plates were inoculated with the same species on both sides of the Petri dishes and incubated under identical conditions. Fungal interactions were recorded and photographed. Each strain combination was tested in 4–6 replicates. Fungal growth was quantified as a percentage relative to the corresponding control, which was set at 100%.

### 2.7 Culture-conditioned medium assay for diffusible extracellular antifungal activity

This assay was used to evaluate the antifungal activity of agar previously conditioned by fungal growth, while excluding direct physical contact and mycoparasitism. Because the assay does not distinguish the effects of diffusible extracellular products from those of nutrient depletion or changes in medium pH, the results were interpreted as the combined effect of fungal culture conditioning. The activity of the tested fungal species (Section 2.6) was assessed using a cellophane-overlay technique (Li et al., 2019). Briefly, sterile cellophane sheets were placed on the surface of PDA plates, and each tested fungal species was grown on the overlaid medium for 2–3 days at 28 ± 1 °C in darkness, until approximately three-quarters of the membrane surface was covered. The cellophane sheets and associated fungal biomass were then carefully removed, leaving fungus-conditioned PDA without fungal biomass. Subsequently, the plates were inoculated centrally with 6-mm agar plugs excised from the actively growing margins of the test fungi. Control plates consisted of PDA overlaid with sterile cellophane, without prior fungal growth, and were otherwise treated identically. Colony diameter was measured after an additional 2 days of incubation at 28 ± 1 °C along two perpendicular axes, and the two measurements were averaged. Each treatment comprised 4–5 independent biological replicates.

### 2.8 Secreted volatile compounds inhibition assay

The volatile-mediated antagonistic effects of the selected root-associated fungal isolates, including *T. asperellum* P1, on *M. phaseolina* were evaluated using a sealed face-to-face “sandwich” plate assay (Li et al., 2018). Briefly, 6-mm mycelial plugs excised from the actively growing margins of 2–4-day-old antagonist fungal colonies were placed at the center of 90-mm PDA plates, while analogous plugs of the pathogen were inoculated onto separate PDA plates. After an initial 24-h growth period, the lids were removed, and the agar surfaces of the “source” (antagonist; lower plate) and “receiver” (pathogen; upper plate) plates were aligned face-to-face and sealed together with two layers of Parafilm to create a shared enclosed headspace without direct physical contact. Pathogen-only plates (incubated inverted) and pathogen–pathogen paired plates served as negative controls. The sealed assemblies were incubated at 28 ± 1 °C in darkness for 1–2 days, until the pathogen in control plates reached approximately two-thirds of the plate radius. At the end of the incubation period, pathogen colony diameters were measured along two perpendicular axes and averaged. Each treatment consisted of five independent biological replicates.

### 2.9 Comparative evaluation of *Trichoderma asperellum* P1 antagonistic activity

The antagonistic activity of *T. asperellum* P1 against *M. phaseolina* was compared across four assay formats. The dual-culture confrontation and volatile-mediated inhibition assays were conducted in the present study as described in Sections 2.6 and 2.8, respectively. Replicate-level data for the shallow stationary cell-free growth-fluid assay and solid-medium growth-fluid assay were obtained from previously published experiments (Degani et al., 2023a). The dataset comprised five replicates for the confrontation assay, five for the volatile-mediated assay, 11 for the shallow growth-fluid assay, and six for the solid-medium growth-fluid assay. For this comparison, *M. phaseolina* growth was evaluated 3 days after inoculation. Representative confrontation plates were additionally photographed after 3 or 6 days, depending on the assay, to document the progression of the interaction. Pathogen inhibition was calculated relative to the corresponding assay-specific control:


Inhibition (%) =[(G_control − G_treatment) / G_control]× 100

where *G_control_* and *G_treatment_* represent M. phaseolina growth, measured as colony diameter or surface coverage according to the assay, in the corresponding control and treatment, respectively. Percentage-inhibition values were compared across assay formats using the Kruskal–Wallis test, followed by multiplicity-adjusted Dunn’s comparisons at *p* < 0.05.

### 2.10 6-Pentyl-α-pyrone antifungal assay

Commercial 6-pentyl-α-pyrone (6-PP; CAS No. 27593-23-3; ≥96% purity; density, 1.004 g mL^-^¹ at 25 °C; Sigma-Aldrich, Rehovot, Israel) was used as supplied. The liquid compound was added directly, without a carrier solvent, to separate 105.13-mL batches of molten PDA cooled to approximately 35 °C. Aliquots of 19.84, 39.70, or 79.40 µL were added to prepare treatment groups nominally designated as 200, 400, and 800 µg mL^-^¹, respectively. Based on the stated product density and PDA volume, these additions corresponded to approximately 189, 379, and 758 µg commercial product mL^-^¹, respectively; the prespecified nominal designations are used throughout the text and figures. The amended media were thoroughly mixed, and 15 mL was poured into each 90-mm Petri dish. Unamended PDA without a carrier solvent served as the control.

The assay included *M. phaseolina* and *M. maydis* isolate Mm2 (CBS 133165; CBS Collection, Westerdijk Fungal Biodiversity Institute, Utrecht, the Netherlands). The latter, a cotton-associated opportunistic fungal pathogen (Degani et al., 2020), was included as a reference strain that had previously been evaluated for its response to 6-PP (Degani et al., 2021). Six independent biological replicates per treatment were included in the quantitative analysis. For each assay, a 6-mm agar disk excised from the margin of an actively growing fungal culture was placed at the center of the plate. Plates were incubated at 28 ± 1 °C in darkness, and colony diameter was measured after 24, 48, and 72 h along two perpendicular axes; the two measurements were averaged. Net mycelial growth was calculated by subtracting the initial 6-mm inoculum-plug diameter from the average colony diameter.

### 2.11 Greenhouse seedling experiment

#### 2.11.1 Experimental setup and inoculation

The experiment was designed to evaluate fungal interactions and *M. phaseolina* root colonization during the early latent stage; it was not intended to assess late-season symptom development or yield effects. The seedling assay included ten treatments, each initially comprising seven independent biological replicates (pots), for a total of 70 pots. Four root-associated fungal isolates—*A. niger* Ed11B, *A. alternata* Ed20A, *R. arrhizus* Ed43A, and *N. solani* Ed68A—were evaluated individually and in combination with *M. phaseolina*. The remaining treatments were an *M. phaseolina*-only infected control and a non-inoculated control. All fungal inoculations were performed twice, at sowing and 13 days after sowing. At each inoculation event, the infected control received 25 *M. phaseolina* culture discs per pot, single-isolate treatments received 25 discs of the corresponding test isolate, and co-inoculation treatments received 25 *M. phaseolina* discs plus 25 discs of the respective test isolate. The 6-mm discs were excised from the actively growing margins of 5–7-day-old colonies, distributed evenly over the soil surface, and buried at approximately 2.5 cm depth. The non-inoculated control received neither fungal culture discs nor sterile culture-medium discs because fresh uncolonized medium could independently affect seed germination and early seedling development. The experiment schedule is detailed in **Table 3**.

**Table 3:** Experimental arrangement of the greenhouse pot trial.

Date	Inoculation and sowing	Days from sowing
08 October 2024	Sowing, 1^st^ inoculation	0
12 October 2024	Aboveground emergence	4
14 October 2024	Seedling emergence assessment 1	6
21 October 2024	2^nd^ inoculation and thinning	13
21 October 2024	Seedling emergence assessment 2	13
21 October 2024	Canopy cover evaluation	13
10 November 2024	Final sampling	33

#### 2.11.2 Growth conditions

The trial was conducted in 2.5-L pots in a greenhouse at the Avnei Etan Experimental Farm, located in the Golan Heights, northeastern Israel (32°49′03.3″ N, 35°45′46.4″ E). The peat soil used in the experiment was obtained from a field with no recorded history of *M. phaseolina* infection; any potential pathogen presence was therefore considered negligible. The soil was amended with 33% (v/v) Perlite No. 4 to improve aeration. Seven seeds of the Goliath V-6 cotton cultivar (*Gossypium barbadense*, extra-long staple Pima-type; Israel Seeds) were sown per pot at an approximate depth of 3 cm and subsequently thinned to five seedlings per pot 13 days after sowing. Plants were maintained under greenhouse conditions with an 11 h light/13 h dark photoperiod, relative humidity ranging from 32% to 100% (mean: 66% ± 19%), and temperatures between 9.8 °C (minimum) and 32.1 °C (maximum), with an average of 21.1 ± 4.3 °C. Pots were irrigated daily using a computerized drip system, which supplied tap water at a flow rate of 2 L·h^-^¹ for 3 min per pot (100 mL day^-^¹). The experiment was terminated 33 days after sowing.

#### 2.11.3 Measurements of traits

Each pot was treated as an independent biological replicate. Measurements obtained from individual plants were averaged within each pot to generate one pot-level value. Endpoint analyses included 6–7 pots per treatment because isolated seedling mortality prevented calculation of a complete final value for one pot in some treatments. All available pot-level data were included, without statistical outlier exclusion. Seedling emergence was assessed 6 and 13 days post-sowing, and plant canopy cover percentage was evaluated 13 days post-sowing. At the end of the experiment (day 33), plant survival and growth parameters, including aboveground fresh weight per plant (shoot weight), shoot length, leaf number, and canopy cover percentage, were recorded. Canopy cover was quantified using Canopeo software (version 2.0; https://canopeoapp.com, accessed 15 January 2026) on a Samsung Galaxy S24 Ultra smartphone (Patrignani and Ochsner, 2015). Images were acquired from a height of 0.5 m above the base of the pot. Pathogen colonization was determined by quantifying *M. phaseolina* DNA levels in cotton roots using quantitative real-time PCR (qPCR), as described in Section 2.12. Minimal qPCR signals were detected in uninoculated control roots, likely reflecting low natural background levels of *M. phaseolina* in the soil.

### 2.12 Analysis of the *M. phaseolina* DNA level

DNA was extracted from roots pooled from all available plants within each pot. The roots were thoroughly washed under running tap water, cut into approximately 2-cm segments, combined within each pot, and 0.7 g of the pooled tissue was used for DNA extraction. Each pot, therefore, yielded one biological DNA sample. Four technical qPCR wells were averaged to generate a single *M. phaseolina* infection estimate per pot, and only these pot-level values were used in the statistical analysis. Fungal DNA extraction was performed following a previously described CTAB-based protocol (Shofman and Degani, 2025). Briefly, 0.7 g of plant tissue was homogenized in 4 mL of CTAB buffer, and a 1.2-mL aliquot was transferred to microcentrifuge tubes and incubated at 65 °C for 20 min. Samples were then centrifuged at 18,000 × g for 5 min at 24 °C, after which 700 µL of the supernatant was extracted with an equal volume of chloroform: isoamyl alcohol (24:1, v/v). This purification step was repeated twice, with 500 µL of each phase used during the second extraction. The aqueous phase (300 µL) was mixed with 200 µL of pre-chilled isopropanol and incubated at −20 °C for 20–60 min to precipitate DNA. DNA was pelleted by centrifugation (12,000 rpm, 4 °C, 20 min), washed with 0.5 mL of ethanol, and air-dried overnight under sterile conditions. The DNA pellet was finally resuspended in 100 µL of ultra-pure water and stored at −20 °C until qPCR analysis.

A previously described qPCR protocol was used to detect *M. phaseolina* using the species-specific MpKFI/MpKRI primer pair (**Table 2**; Degani et al., 2023a). To account for variation in DNA extraction and sample amplifiability, the mitochondrial cytochrome *c* oxidase (COX) primer pair (**Table 2**) was used as a broad plant mitochondrial internal control (Weller et al., 2000; Li et al., 2006). This primer pair is not cotton-specific, and uninoculated cotton-root samples were included as plant controls. Because pure-culture DNA from the other inoculated fungi was not tested separately with the COX primers, possible fungal cross-amplification cannot be completely excluded. Relative *M. phaseolina* DNA signals were calculated using the ΔCt (delta cycle threshold) method, assuming comparable amplification efficiencies for the target and reference assays. Raw COX Ct values were analyzed across treatments using the Kruskal–Wallis test followed by Dunn’s multiple-comparison test to assess potential variation in internal-control amplification.

All qPCR assays were conducted with four technical replicates using a CFX384 qPCR Detection System (Bio-Rad, Hercules, CA) in 384-well plates. Each 5-µL reaction contained 0.25 µL each of forward and reverse primers (5 µmol L^-^¹), 2.5 µL of iTaq™ Universal SYBR Green Supermix (Bio-Rad Laboratories), and 2 µL of diluted DNA template. Thermal cycling conditions consisted of an initial activation step at 95 °C for 60 s, followed by 40 cycles of denaturation at 95 °C for 15 s and annealing/extension at 60 °C for 30 s, with a final melting-curve analysis (Degani et al., 2023a).

### 2.13 Statistical analysis

Statistical analyses were performed using GraphPad Prism version 10.4.2 (GraphPad Software Inc., San Diego, CA). The individual plate was the biological replicate in the *in vitro* assays, and the individual pot was the biological replicate in the greenhouse experiment. Plant measurements were averaged within each pot. For qPCR, roots from all available plants in each pot were pooled before DNA extraction, and technical-well values were averaged to obtain one pot-level value. All available biological replicate data were included without excluding statistical outliers.

Residual normality and homogeneity of variance were evaluated using the Shapiro–Wilk and Brown–Forsythe tests, respectively. Data that satisfied both assumptions were analyzed using an ordinary one-way ANOVA. Normally distributed data with unequal variances were analyzed by Welch’s ANOVA, whereas nonnormally distributed data were analyzed by the Kruskal–Wallis test. Where applicable, pairwise comparisons were performed using Tukey’s test after ordinary ANOVA, Dunnett’s T3 test after Welch’s ANOVA, or Dunn’s test after Kruskal–Wallis analysis. Pairwise *P* values were adjusted for multiple comparisons, and statistical significance was set at *p* < 0.05.

For the 6-pentyl-α-pyrone assay, each fungal species was analyzed separately using the six plates per treatment, measured at all three time points. Net mycelial growth was analyzed using a two-way mixed-design repeated-measures ANOVA, with nominal 6-PP concentration as the between-plate factor and measurement time as the within-plate factor. Sphericity was not assumed, and the Greenhouse–Geisser correction was applied to effects involving time. For *M. phaseolina*, Dunnett’s multiple-comparison test was used to compare each concentration with the corresponding time-matched PDA control. For *M. maydis*, the 400 µg mL^-^¹ treatment was compared with its corresponding time-matched control using Šídák’s multiple-comparison test. Statistical significance was defined as *p* < 0.05.

Effect sizes are reported as η² for parametric analyses and ϵ² for Kruskal–Wallis analyses. Both range from 0 to 1, with larger values indicating a stronger association between treatment and the measured outcome. Observed values in this study, η² ranged from 0.126 to 0.956 and ε² from 0.000 to 0.887. Assumption-test results, omnibus statistics, degrees of freedom, exact *P* values, effect sizes, and *post hoc* procedures are provided in **Supplementary Table S5**. The field culture-recovery summaries were treated descriptively and were not subjected to analyses of overall fungal community composition or diversity.

## 3 Results

This study examined culturable root-associated microorganisms recovered from cotton roots under high CRD pressure, using a culture-dependent isolation approach to assess the effects of chemical, biological, and integrated management treatments. Interactions among selected root-associated fungal taxa were further explored using *in vitro* and greenhouse assays.

### 3.1 Isolation and identification of culturable cotton root-associated microorganisms

From 168 root samples collected during the growing season, 66 representative isolates were selected and molecularly identified (**Table 1**). Isolates were identified by ITS molecular profiling (**Supplementary Table S2**) and phylogenetic analysis, supported by ICN-recognized type strains for each genus and species (**Figures 1**–**4**). Bidirectional ITS4/ITS5 BLASTN and Fusarioid-ID analyses showed 80.34–100% sequence similarity to GenBank entries, with ≥98% similarity in most cases. The recovered microorganisms included 10 bacterial and 56 fungal isolates, classified into 8 bacterial species (5 genera) and 24 fungal species (11 genera).

Phylogenetic analysis of the ITS region revealed distinct clustering patterns that supported the taxonomic identities of the cotton root fungal isolates. The isolates grouped into major clades corresponding to *Talaromyces*, *Aspergillus*, *Penicillium*, *Chaetomium*, *Gibellulopsis*/*Gloeotinia*, *Trichoderma*, *Fusarium*/*Neocosmospora*, *Rhizopus*, and *Alternaria*. Within each genus, isolates generally clustered with their nearest GenBank reference or type strains, supporting genus- or species-level assignments. The overall tree topology showed clear separation among major fungal groups and strong similarity within several species-level clusters. These results indicate that the culture-recovered cotton root-associated fungal collection in the sampled fields included multiple well-characterized fungal taxa, alongside several isolates showing moderate divergence consistent with intraspecific variability or lower-confidence species assignment. A complementary clustering tree based on ITS5 sequences showed a comparable pattern (**Supplementary Figure S1**).

The proportion of each fungal genus among the total species or isolates identified at each sampling point during the cotton growth season is presented in **Figure 5**. The comparison highlights temporal changes in the relative representation of recovered fungal genera associated with sampling date and visual symptom status, showing an increased representation of pathogenic and opportunistic genera during plant maturation, with a pronounced enrichment in roots of symptomatic plants at the season’s end. Throughout the season, *Aspergillus* was the most frequently represented genus in the culture-recovered fungal collection from asymptomatic roots (30–43%), followed by *Trichoderma* (14–20%). In contrast, the relative proportion of other previously dominant genera, such as *Rhizopus*, declined concomitantly over time with the emergence of additional taxa (e.g., *Alternaria*). At the end of the season, Alternaria was the most frequently recovered genus in symptomatic roots, accounting for 35% of fungal isolates (**Figure 5F**). However, it represented only 6% of the distinct fungal taxa in this group (**Figure 5C**). Notably, *T. asperellum* was recovered from two root samples collected from plants receiving only chemical treatment and no biological seed coating (**Supplementary Table S2**), suggesting that this species was naturally present at the field site. However, because strain-level identity was not established, these isolates could not be definitively identified as the P1 strain.

**Figure 5:**
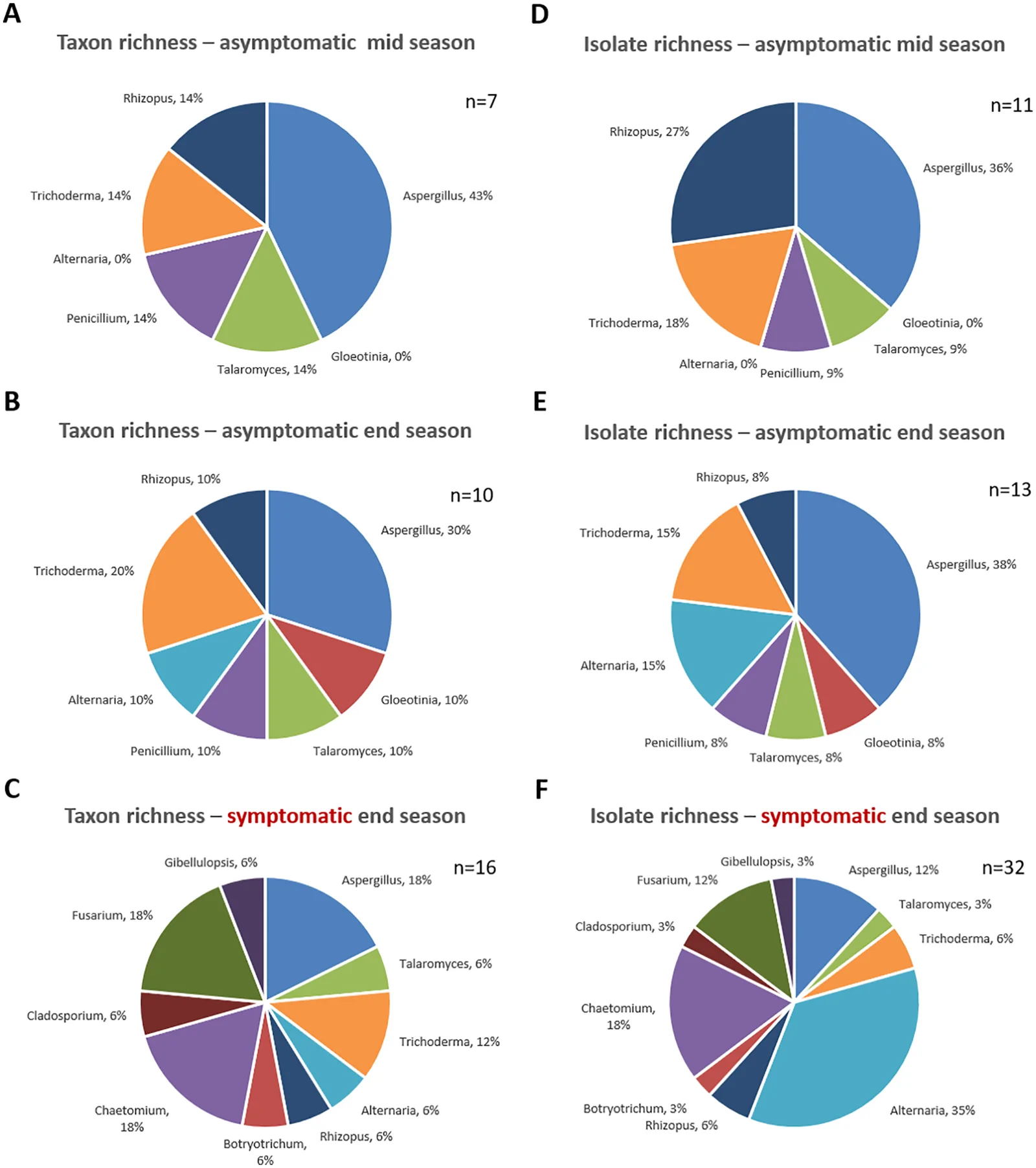
Distribution of molecularly identified representative fungal isolates recovered from cotton roots across visual symptom categories and sampling times. Pie charts illustrate the relative proportions of recovered fungal genera based on taxon richness within the culture-recovered collection [number of distinct fungal taxa; **(A–C)**] and the number of recovered fungal isolates **(D–F)**. Data are shown for asymptomatic plants at mid-season **(A, D)**, asymptomatic **(B, E)**, and symptomatic plants **(C, F)** at the end of the season. Percentages represent the proportion of each fungal genus among the total species or isolates identified at each sampling point. In panels **(C, F)**, the category labeled “Fusarium” also includes isolates identified as Neocosmospora solani.

Comparison among management practices applied against the CRD pathogen revealed a distinct pattern (**Figure 6**). The highest taxon richness within the culture-recovered fungal collection was consistently observed throughout the growing season in plants subjected to integrated (biological and chemical; 43–55%) or chemical (29–38%) management, with a slight increase over time. In contrast, under *Trichoderma* seed-coating treatment, taxon richness within the culture-recovered fungal collection declined markedly, from 29% to 9% in asymptomatic plants and to 15% in symptomatic plants.

**Figure 6:**
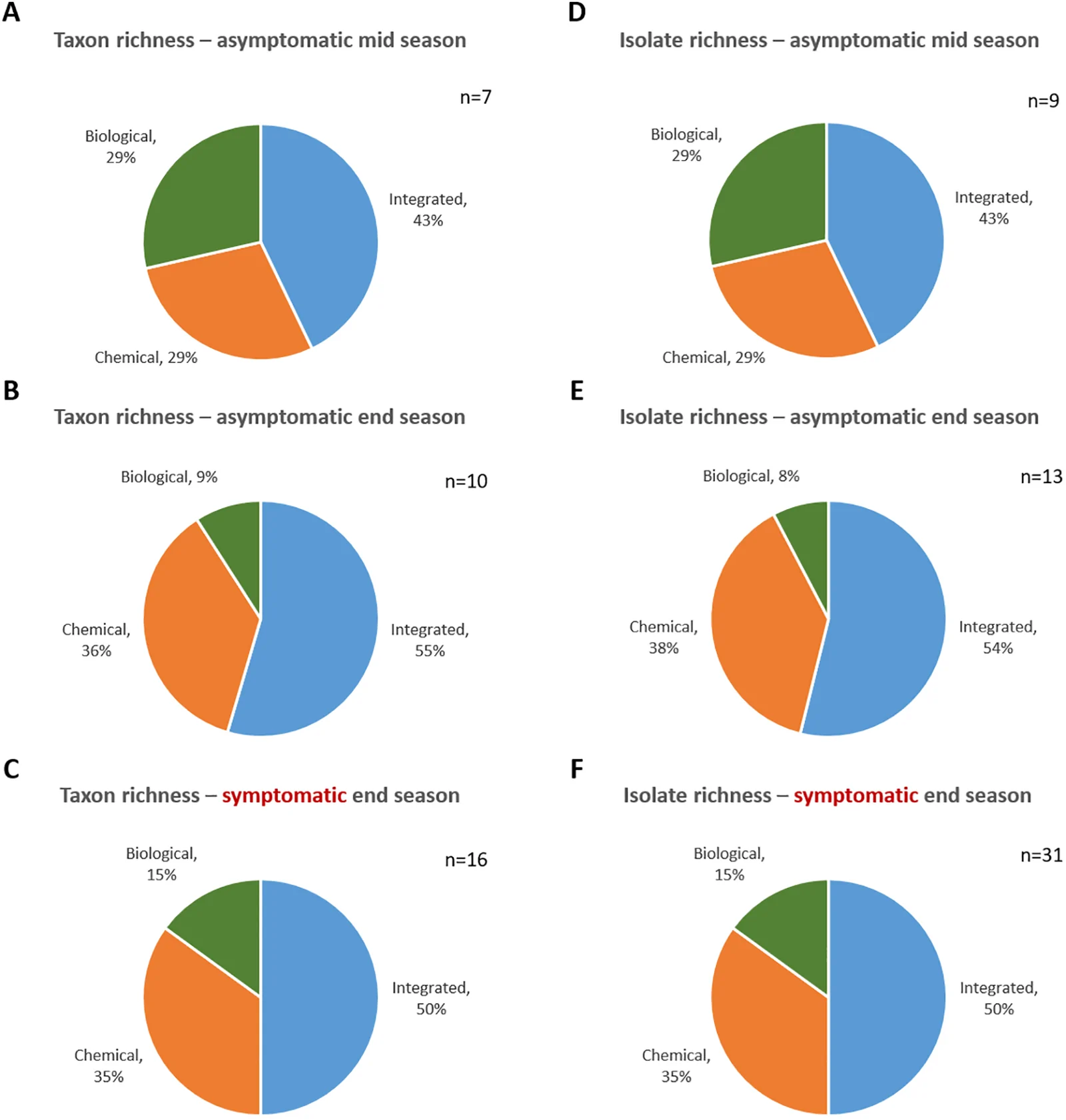
Patterns of molecularly identified representative fungal isolates under different disease-management practices. Pie charts compare the relative proportions of fungi recovered using culture-dependent methods under integrated (biological + chemical), chemical, and biological (*Trichoderma* seed-coating) management practices, based on taxon richness within the culture-recovered collection [number of distinct fungal taxa; **(A–C)**] and the number of recovered fungal isolates **(D–F)**. Data are presented for asymptomatic plants at mid-season **(A, D)**, asymptomatic **(B, E)**, and symptomatic plants **(C, F)** at the end of the season. Percentages indicate the proportion of total species or isolates associated with each management regime at each sampling point.

### 3.2 Plate confrontation assay

Interactions among selected cotton root-associated fungi were examined using dual-culture plate assays (**Figure 7**; **Supplementary Figure S2**). The growth of *M. phaseolina* was differentially affected by co-cultivation with other fungi; *A. alternata* (Ed20A) induced a 27% increase in colony growth, whereas *R. arrhizus* (Ed43A) exerted a moderate suppressive effect (25%; *p* < 0.05; **Figure 7**). In contrast, the growth of *A. niger* (Ed11B), *A. alternata*, and *N. solani* (Ed68A) was markedly reduced in the presence of competing fungi, with mean relative growth of approximately 70%, 76%, and 66% of the corresponding controls, respectively, indicating potential interspecific antagonism among the selected root-associated fungi (**Figures 7B, C, E**). Conversely, *R. arrhizus* exhibited pronounced growth stimulation (49–65%, *p* < 0.05) when challenged with most tested pathogens, except *M. phaseolina*, which had no significant effect on its growth (**Figure 7D**). This rapidly growing species overgrew the colonies of *M. phaseolina* and *N. solani*, covering nearly the entire colony surface within 6 days (**Supplementary Figure S2**).

**Figure 7:**
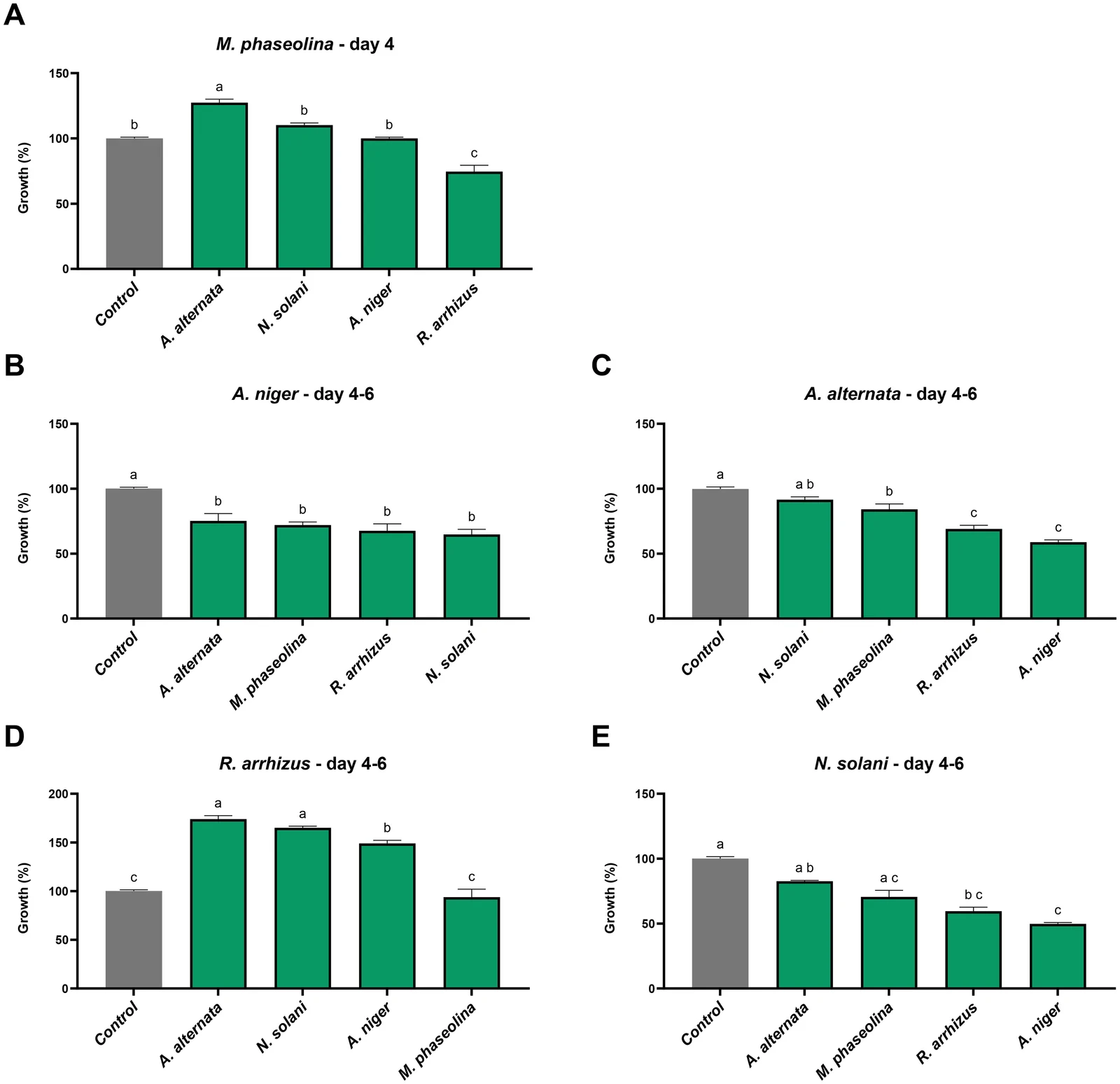
Plate confrontation assay evaluating interactions among selected cotton root-associated fungal isolates and *M. phaseolina* (**Supplementary Table S2**). Colony growth was expressed as a percentage of the corresponding dual-culture control and quantified after 4–6 days of incubation. Panels show the responses of **(A)** *Macrophomina phaseolina*, **(B)** *Aspergillus niger*, **(C)** *Alternaria alternata*, **(D)** *Rhizopus arrhizus*, and **(E)** *Neocosmospora solani* when paired with the other test fungi. Bars represent means ± SE based on 4–6 biological replicates per treatment. Differences among treatments were evaluated using one-way ANOVA followed by Tukey’s test in panels **(A–C)**, Welch’s ANOVA followed by Dunnett’s T3 in panel **(D)**, and the Kruskal–Wallis test followed by Dunn’s test in panel **(E)**. *Post hoc p* values were adjusted for multiple comparisons. Different lowercase letters indicate significant differences among treatments (*p* < 0.05). Complete statistical results, including assumption tests, omnibus *P* values, and effect sizes, are provided in **Supplementary Table S5**.

Similar to the plate confrontation assay, the diffusible extracellular activity represents an additional important antagonistic mode of action, differentiating among tested root-associated fungi (**Figure 8**). In this assay, growth inhibition was nearly twofold higher than in the dual-culture assay, reaching 24–66% suppression when *A. niger* antagonized the other species (except *R. arrhizus*). Another strong antagonist was *N. solani*, which displayed pronounced inhibitory activity against *M. phaseolina* (74%), *A. niger* (56%), and *A. alternata* (32%), in marked contrast to its negligible effect in the direct confrontation assay with *M. phaseolina* and *A. alternata* (**Figure 7**). An exception was *R. arrhizus*, which, consistent with the plate confrontation assay, showed enhanced growth when exposed to diffusible compounds from the other tested species.

**Figure 8:**
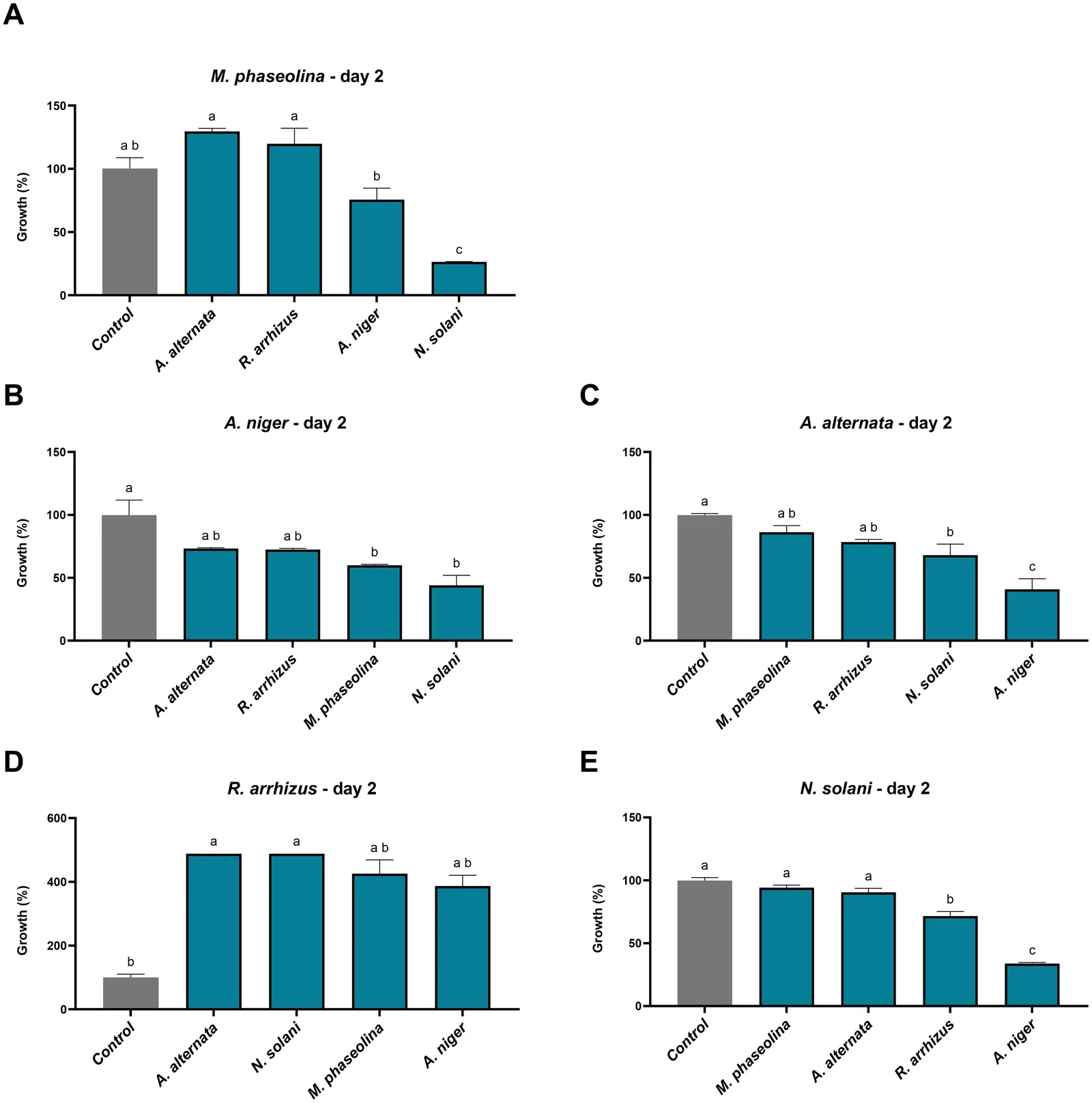
Culture-conditioned medium assay evaluating diffusible extracellular antifungal activity among selected root-associated fungal species (**Supplementary Table S2**). Relative colony growth (%) was quantified against the corresponding unconditioned-medium control after 2 days of incubation. Panels show the responses of **(A)** *Macrophomina phaseolina*, **(B)** *Aspergillus niger*, **(C)** *Alternaria alternata*, **(D)** *Rhizopus arrhizus*, and **(E)** *Neocosmospora solani*. Bars represent means of 4–5 biological replicates ± SE. Different lowercase letters indicate significant differences among treatments within each panel (*p* < 0.05). Panels **(A, C, E)** were analyzed using one-way ANOVA followed by Tukey’s *post hoc* test, whereas panels **(B, D)** were analyzed using the Kruskal–Wallis test followed by Dunn’s multiple-comparison test. *Post hoc p*-values were adjusted for multiple comparisons. Complete test statistics, omnibus *p*-values, assumption checks, and effect sizes are provided in **Supplementary Table S5**.

Exposure to fungal volatile metabolites resulted in generally weak, species-specific growth responses among the tested cotton root–associated fungi (**Figure 9**). *M. phaseolina* growth was significantly reduced only by volatiles produced by *R. arrhizus*. Aspergillus niger showed numerically lower growth when exposed to volatiles from M. phaseolina or R. arrhizus, but neither treatment differed significantly from the control (**Figure 9B**). In most other interactions, exposure to volatiles was associated with unchanged or increased growth, consistent with tolerance to airborne metabolites.

**Figure 9:**
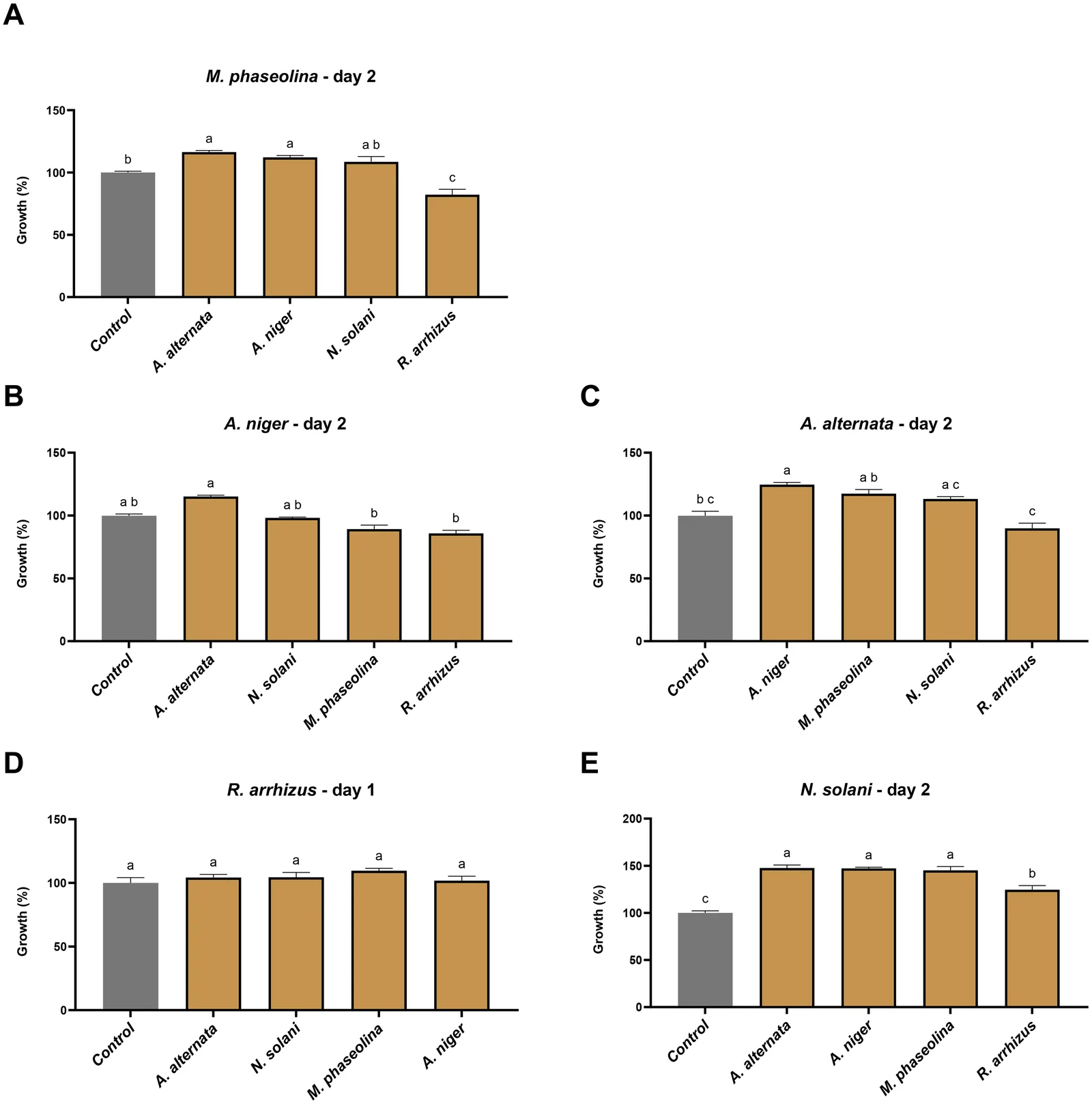
Volatile-mediated interactions among selected cotton root-associated fungal isolates. Colony growth, expressed as relative colony area (%), was quantified after 1 day for *Rhizopus arrhizus* **(D)** and after 2 days for the other species, relative to the corresponding control cultures. Panels show the response of **(A)** *Macrophomina phaseolina*, **(B)** *Aspergillus niger*, **(C)** *Alternaria alternata*, **(D)** *R. arrhizus*, and **(E)** *Neocosmospora solani* to volatile compounds produced by the other tested fungi in the sealed dual-plate assay. Bars represent mean values ± SE of five biological replicates. Different lowercase letters indicate significant differences among treatments based on multiplicity-adjusted *p* values (*p* < 0.05). Panels **(A, D, E)** were analyzed by one-way ANOVA followed by Tukey’s *post hoc* test, whereas panels B and C were analyzed by the Kruskal–Wallis test followed by Dunn’s multiple-comparison test.

The field seed-coating formulation included *Trichoderma asperellum* P1 and related *Trichoderma* spp. previously shown to suppress *M. phaseolina* effectively (Degani et al., 2024b). Comparative *in vitro* evaluation of P1 revealed pronounced differences among the four assay formats (**Figure 10**). Direct confrontation produced the highest mean inhibition of *M. phaseolina* growth (55%), followed by volatile-mediated inhibition (51%) and the shallow growth-fluid assay (38%); these three assay formats did not differ significantly (*p* > 0.05). In contrast, the solid-medium growth-fluid assay resulted in negligible inhibition (3%) and was significantly less effective than the other formats (*p* < 0.05). Representative plate images supported these findings, showing progressive overgrowth of *M. phaseolina* by P1 between days 3 and 6 and marked restriction of pathogen growth in the shallow growth-fluid assay, whereas growth on the solid-medium preparation was similar to that of the corresponding control. The progressive overgrowth observed during direct confrontation was consistent with mycoparasitic activity.

**Figure 10:**
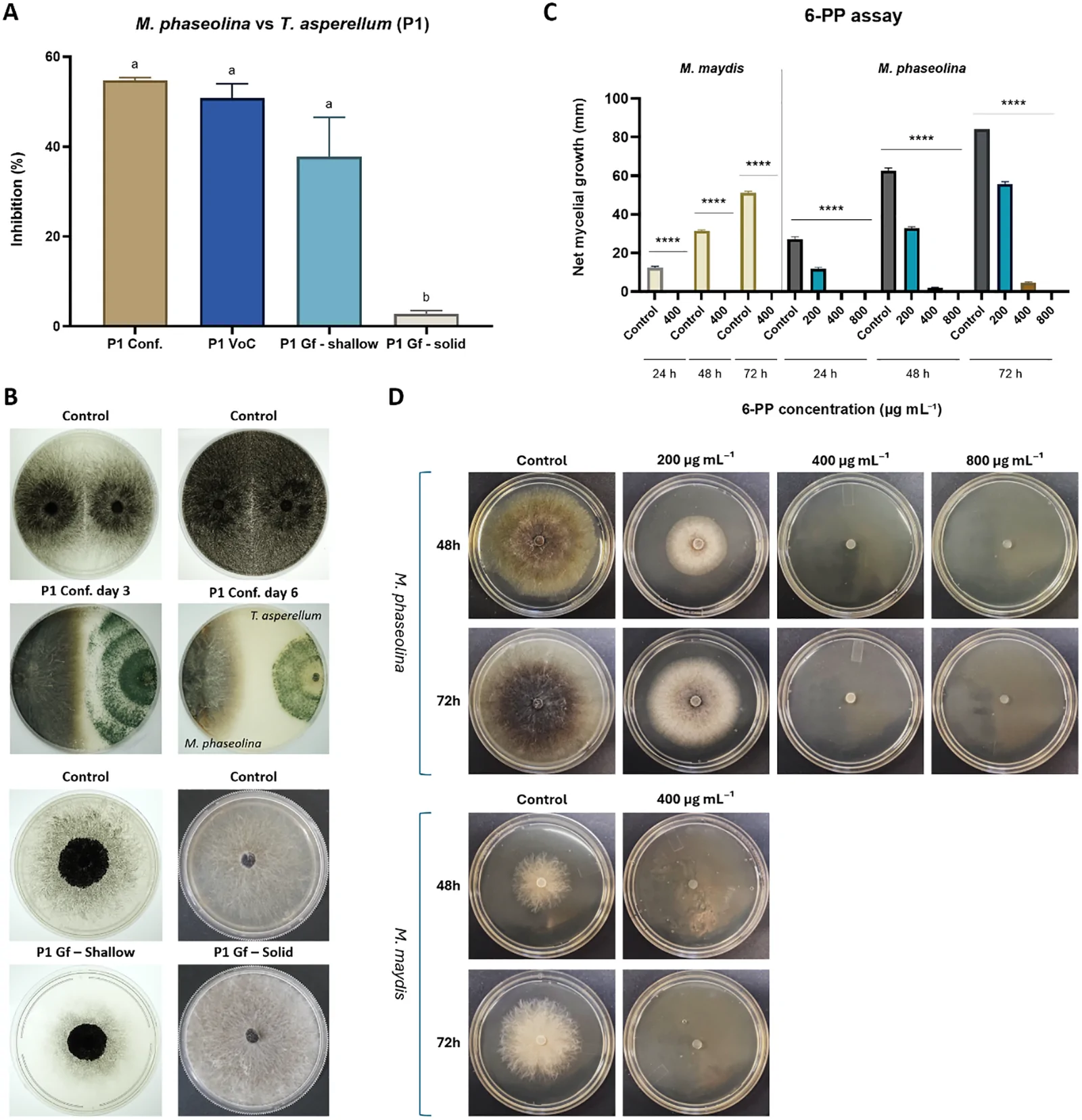
*In vitro* antifungal activity of *Trichoderma asperellum* P1 and 6-pentyl-α-pyrone (6-PP). **(A)** Inhibition of *Macrophomina phaseolina* growth in four assay formats: dual-culture confrontation (P1 Conf., *n* = 5), volatile-mediated inhibition (P1 VOC, *n* = 5), a shallow stationary liquid-culture assay using cell-free growth fluid (P1 GF-Shallow, *n* = 11; Degani et al., 2023a), and a solid-medium growth-fluid assay (P1 GF - Solid, *n* = 6; Degani et al., 2023a). Assays were conducted at 28 ± 1 °C in darkness, and inhibition was evaluated after 3 days. Bars represent means ± SE. Differences among assay formats were significant according to the Kruskal–Wallis test (*H*(3) = 15.01, approximate *P* = 0.0018, ϵ² = 0.5222), followed by Dunn’s multiple-comparison test. Different lowercase letters indicate significant pairwise differences based on multiplicity-adjusted *P* values (*P* < 0.05). **(B)** Representative images of the assays presented in panel **(A)**. The upper image pairs show the respective controls and P1 dual-culture confrontations after 3 and 6 days. The lower image pairs show the respective controls and P1 treatments in the shallow and solid-medium growth-fluid assays after 3 days. **(C)** Inhibitory activity of commercial 6-PP incorporated into PDA at the indicated nominal concentrations. *Magnaporthiopsis maydis*, previously used to characterize the activity of P1-derived 6-PP (Degani et al., 2021), was included as a reference fungus. Net mycelial growth, calculated by subtracting the initial 6-mm inoculum-plug diameter from the colony diameter, was measured after 24, 48, and 72 h. Bars represent means ± SE of six independent plates followed over time. For *M. maydis*, two-way repeated-measures ANOVA with Greenhouse–Geisser correction revealed significant effects of 6-PP treatment [*F*(1, 10) = 23,768], time [*F*(1.528, 15.28) = 5,360], and their interaction [*F*(1.528, 15.28) = 5,360]. For *M. phaseolina*, significant effects were detected for 6-PP concentration [*F*(3, 20) = 11,796], time [*F*(1.667, 33.35) = 14,689], and their interaction [*F*(5.002, 33.35) = 4,322]. All omnibus effects had *P* < 0.0001. Šídák’s test was used to compare the *M. maydis* treatment with its corresponding time-matched control, whereas Dunnett’s test was used to compare each 6-PP concentration with the corresponding *M. phaseolina* control. At each time point, **** indicates that all displayed treatment–control comparisons were significant after multiplicity adjustment (adjusted *P* < 0.0001). **(D)** Representative plates from the 6-PP assay after 48 and 72 h. The upper image set shows *M. phaseolina* grown on PDA containing 0, 200, 400, or 800 µg mL^-^¹ 6-PP, whereas the lower image set shows *M. maydis* grown on PDA containing 0 or 400 µg mL^-^¹ 6-PP.

The *M. maydis* isolate, an opportunistic fungal pathogen associated with cotton, was included as a reference strain previously evaluated for its response to 6-PP (Degani et al., 2021). Overall, 6-PP markedly suppressed the net mycelial growth of both fungal species at all time points examined (adjusted P < 0.0001; **Figures 10C, D**). For *M. phaseolina*, significant effects of 6-PP concentration, incubation time, and their interaction were detected (*p* < 0.0001), indicating that treatment efficacy was both concentration- and time-dependent. At 200 µg mL^-^¹, growth inhibition decreased from 56.5% at 24 h to 33.8% at 72 h, whereas 400 µg mL^-^¹ maintained 94.5–100% inhibition and 800 µg mL^-^¹ completely suppressed measurable growth throughout the experiment. For *M. maydis*, significant effects of 6-PP treatment, incubation time, and their interaction were also observed (*P* < 0.0001). The 400 µg mL^-^¹ treatment completely suppressed measurable mycelial growth at 24, 48, and 72 h, whereas growth in the corresponding controls increased progressively over time.

### 3.3 Greenhouse seedling experiment

Interactions were further examined in planta by evaluating *M. phaseolina* in the presence of representative root-associated pathogenic fungi from major culture-recovered root-associated genera. Because interactions among co-colonizing root fungi remain poorly characterized, inoculations were applied at sowing and repeated 13 days later to assess early seedling development (**Figure 11**) and *M. phaseolina* root colonization by qPCR (**Figure 12**). Raw COX Ct values differed overall among treatments (Kruskal–Wallis, *H*(9) = 20.09, approximate *p* = 0.0173, ϵ² = 0.1981; **Supplementary Table S5**). However, multiplicity-adjusted Dunn comparisons detected no significant difference between any fungal treatment and the *M. phaseolina*-only control (*p* > 0.05).

**Figure 11:**
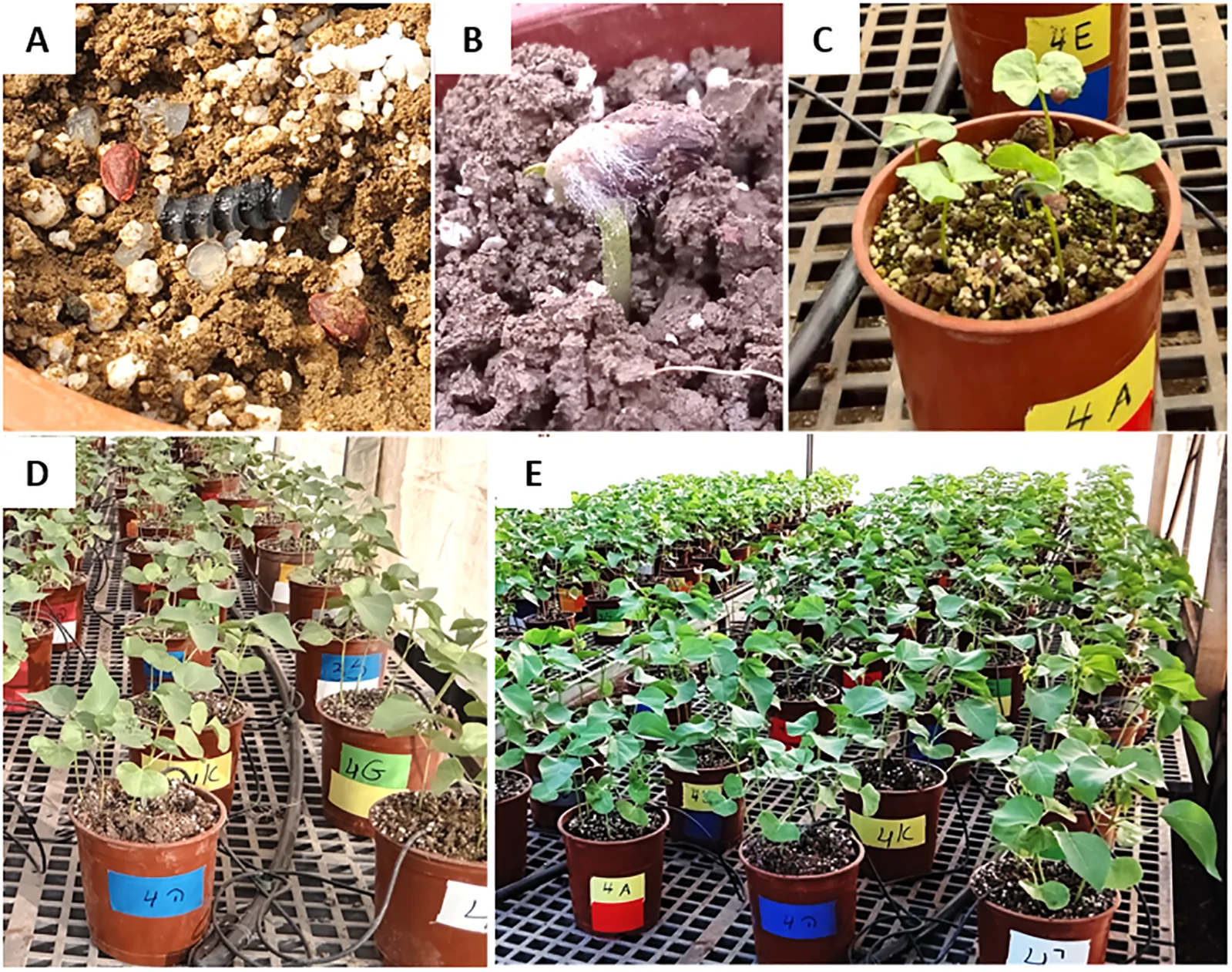
Experimental setup and seedling development in the greenhouse assay used to evaluate interactions between *Macrophomina phaseolina* and representative root-associated fungi. **(A)** Initial culture-disc inoculation and cotton-seed placement at sowing. All fungal treatments were applied again 13 days after sowing; the second inoculation is not shown. **(B)** Colonization of the seed coat by *Rhizopus arrhizus* Ed43A at 5 days after sowing. Representative seedling development is shown at 8 **(C)**, 23 **(D)**, and 33 **(E)** days after sowing.

**Figure 12:**
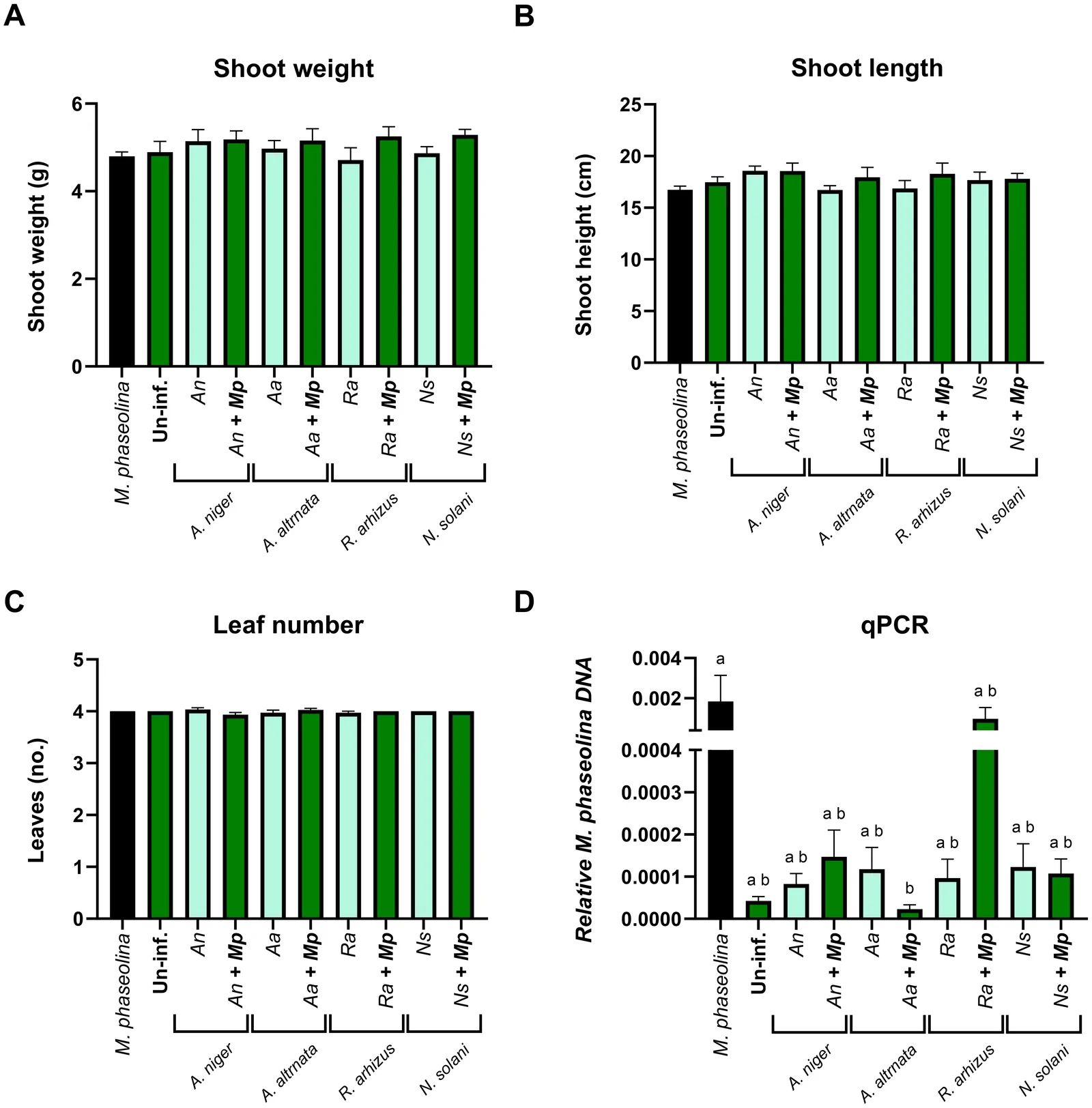
Greenhouse assay of early fungal interactions and *Macrophomina phaseolina* root colonization. The ten treatments comprised a non-inoculated control (Un-inf.), an *M. phaseolina*-only infected control, each selected fungus applied alone, and each fungus co-inoculated with *M. phaseolina*: *Aspergillus niger* (*An*), *Alternaria alternata* (*Aa*), *Rhizopus arrhizus* (*Ra*), and *Neocosmospora solani* (*Ns*). Shoot weight **(A)**, shoot length **(B)**, leaf number **(C)**, and relative *M. phaseolina* DNA in the roots determined by qPCR **(D)** were assessed 33 days after sowing. Values represent means ± SE of 6–7 independent pots per treatment. All available pot-level observations were included without statistical outlier exclusion. For qPCR, roots from all available plants within each pot were pooled before DNA extraction, and technical qPCR wells were averaged to generate one value per pot. Panels A and B were analyzed by one-way ANOVA followed by Tukey’s multiple-comparisons test: **(A)** *F*(9, 56) = 0.9126, *p* = 0.5211, η² = 0.1279; **(B)** *F*(9, 56) = 1.054, *p* = 0.4107, η² = 0.1448. Panels **(C, D)** were analyzed by Kruskal–Wallis tests followed by Dunn’s multiple-comparisons test: **(C)** *H*(9) = 9.946, approximate *p* = 0.3549, ϵ² = 0.0169; **(D)** *H*(9) = 17.74, approximate *p* = 0.0384, ϵ² = 0.1561. *Post hoc p*-values were adjusted for multiple comparisons. Different lowercase letters indicate statistically significant pairwise differences (*p* < 0.05).

Greenhouse evaluation of seedling emergence and early canopy development showed no adverse effects of single or dual fungal inoculations. All treatments, including non-inoculated controls, *M. phaseolina* alone, and co-inoculations with *A. niger*, *A. alternata, R. arrhizu*s, or *N. sola*ni, exhibited uniform emergence at 6 and 13 days after sowing, with mean emergence scores near the maximum score (**Supplementary Table S4**). Canopy cover at 13 days also showed no significant differences among treatments, although co-inoculation with *M. phaseolina and A. ni*ger o*r R. arrhizus* resulted in moderate increases in canopy cover (34% and 22%, respectively) relative to the infected control. A comparable enhancement (33%) was observed in plants inoculated with *A. alternata* alone.

By 33 days after sowing, canopy cover did not differ significantly among treatments (one-way ANOVA, F(9, 56) = 0.894, p = 0.5366, η² = 0.126). Plants inoculated with *A. niger* alone exhibited the highest numerical canopy coverage (15% above the infected control), while *R. arrhizus* alone resulted in reduced canopy development. Dual inoculation with *M. phaseolina* and *N. solani* produced the second-highest increase in canopy cover (12%).

At the end of the experiment, plant development appeared generally normal and was minimally affected by fungal treatments (**Figures 11**, **12**). As expected, *M. phaseolina* did not induce visible symptoms at this latent stage (**Figures 11C–E**). Relative *M. phaseolina* DNA differed significantly among treatments (Kruskal–Wallis, *H*(9) = 17.74, approximate *p* = 0.0384, ϵ² = 0.156). Relative to the mean of the *M. phaseolina*-only control, co-inoculation with A. niger, N. solani, and A. alternata was associated with mean reductions of 92.0%, 94.2%, and 98.8%, respectively, compared with 46.8% for *R. arrhizus*. However, Dunn’s multiple-comparisons test identified a significant difference only between the *M. phaseolina*-only and *A. alternata* + *M. phaseolina* treatments (adjusted *p* = 0.0322); all other adjusted pairwise comparisons were nonsignificant.

## 4 Discussion

This study provides an integrated characterization of the temporal patterns within the culture-recovered root-associated fungal collection under Israeli commercial field conditions by combining culture-dependent profiling with *in vitro* interaction assays and greenhouse functional testing. By linking temporal sampling with visual symptom status and management regimes, the study identified associations between these factors and patterns in the culture-recovered fungal collection. Controlled assays subsequently identified fungal interactions associated with *M. phaseolina* growth and early root colonization. The commercial field trial from which these samples were collected previously demonstrated that *Trichoderma*-based seed coating, applied alone or integrated with low-dose azoxystrobin, provided CRD control comparable to conventional chemical management (Degani et al., 2024b). This biological intervention reduced pathogen root colonization, improved plant health, and maintained yield, with bio-coating combined with fungicide application at sowing showing particular promise.

Culture-dependent profiling revealed pronounced temporal changes in the culture-recovered root-associated fungal collection throughout the growing season. Within the culture-recovered collection, *Aspergillus*, *Rhizopus*, and *Trichoderma* were most frequently represented in mid-season roots during the latent disease stage. In contrast, Alternaria, Fusarium, and Chaetomium were more frequently recovered at season’s end, particularly from symptomatic plants. This progressive diversification is consistent with the notion that plant maturation and declining host vigor increase niche availability for secondary colonists, including latent pathogens and saprotrophs (Xiong et al., 2021; Wang et al., 2022).

The sharp increase in the numbers of recovered fungal species and isolates in symptomatic plants toward the end of the season may be associated with plant development, disease progression, or secondary colonization. The result could also align with emerging paradigms of plant disease in which disease severity reflects cumulative microbial interactions and community dynamics rather than the action of a single dominant pathogen (Mannaa and Seo, 2021). As demonstrated here, disease-management regimes were associated with distinct patterns in the culture-recovered cotton root-associated fungal collection across the two sampling times examined. Chemical and integrated (chemical + biological) treatments were associated with relatively high taxon richness within the culture-recovered fungal collection throughout the season, likely reflecting partial suppression of dominant taxa while preserving ecological space for subdominant colonists (Vannier et al., 2019). In contrast, *Trichoderma* seed coating was associated with lower recovery of fungal taxa at the end of the season. Because the data were culture-dependent and morphotype-selected, longitudinal sequencing studies are needed to determine whether this pattern reflects changes in community diversity, function, or resilience.

Although the reduced fungal recovery under *Trichoderma*-based management may reflect suppression of active or opportunistic pathogens (Baird et al., 2003), it raises the ecologically important question of whether broad biological interventions also affect non-target root-associated fungi. Determining whether overall community richness was altered will require sequencing-based validation. Thus, biocontrol disease management should consider not only pathogen suppression but also the long-term stability and resilience of the root microbial ecosystem (Moussa and Iasur Kruh, 2025). Indeed, recent studies indicate that agricultural practices and disease-control strategies can markedly influence plant microbiome structure and function, with both pesticide applications and biological control agents capable of reshaping phytobiota richness and potentially suppressing beneficial microbial activities (Sangiorgio et al., 2022). Despite this accumulating evidence, the broader impacts of disease-management interventions on plant-associated microbiomes and their downstream consequences for plant growth, crop productivity, and quality remain insufficiently explored.

The confrontation, soluble metabolite, and volatile assays collectively demonstrate that antagonism among the selected root-associated fungi is highly species-dependent and strongly context-specific. Direct dual-culture assays identified *A. niger* and *N. solani* as potent competitors, whereas *R. arrhizus* consistently exhibited growth stimulation or neutral interactions, including physical overgrowth without effective pathogen suppression. Notably, diffusible extracellular activity exerted stronger inhibitory effects than direct contact in several species combinations, underscoring the importance of diffusible antifungal compounds in shaping belowground competition (Degani and Gordani, 2022; Chen et al., 2024). However, metabolite-mediated antagonism observed *in vitro* does not always reflect microbial interactions *in situ* (Köhl et al., 2019). Volatile-mediated antagonism, in contrast, was comparatively weak and inconsistent, highlighting the need for further investigation of the role of airborne signaling in the dense, hydrated root environment.

In the P1 assays, the weak activity of the solid-medium growth-fluid preparation (3% inhibition), compared with the shallow cell-free liquid assay (38%), may reflect differences in the availability and stability of antifungal products between the two assay matrices. Incorporation into a solid medium may dilute, immobilize, or restrict the diffusion of active compounds, whereas the shallow liquid format allows more direct and uniform exposure of the pathogen. Differences in nutrient availability or pH may also have contributed. Therefore, the weak inhibition in the solid-medium assay does not necessarily indicate an absence of active extracellular products but suggests that their detectable activity is strongly dependent on the assay conditions.

Recent reviews emphasize that microbial natural products, including soluble secondary metabolites and volatile organic compounds, can mediate antibiosis, mycoparasitism, plant defense priming, root development, and microbiome-level communication in plant–fungus and microbe–microbe interactions (Yu et al., 2021; Zhao et al., 2023; Montejano-Ramírez et al., 2024; Reveglia et al., 2024; Jin and Alberti, 2025). From a chemical ecology perspective, the stronger inhibition observed in the diffusible extracellular antifungal activity assay than in direct confrontation could indicate that natural products released by root-associated fungi may have contributed *to M. phaseolina* suppression. These diffusible compounds might include organic acids, polyketide- or peptide-derived metabolites, siderophore-related products, and other low-molecular-weight antifungal molecules that alter local pH, nutrient availability, membrane integrity, or hyphal signaling. Such effects can be especially relevant in the rhizosphere, where metabolites released at subinhibitory concentrations can act not only as toxins but also as chemical signals that shift competitive hierarchies and modulate community composition (Li et al., 2019; Chen et al., 2024). The apparent difference between soluble and volatile assays further suggests that, under the hydrated and spatially constrained conditions typical of cotton roots, diffusible metabolites may be more influential than headspace volatiles.

Previous work with the same *T. asperellum* P1 strain demonstrated its capacity to produce 6-pentyl-α-pyrone (6-PP), which was isolated from the culture medium, identified using LC–MS and GC–MS, and shown to inhibit *M. maydis* (Degani et al., 2021). The activity of commercial 6-PP observed here supports its role as a plausible contributor to P1 antifungal activity and extends evidence of its activity to *M. phaseolina*. Exploratory linear interpolation of the *M. phaseolina* data (based on nominal concentrations) yielded apparent EC_50_ values of approximately 177, 211, and 253 µg mL^-^¹ at 24, 48, and 72 h, respectively, consistent with reduced efficacy at lower concentrations over time. These estimates remain provisional because only three nonzero concentrations were tested, and the 24-h EC_50_ was not bracketed by two treated concentrations; a denser concentration series is required for formal nonlinear dose–response modeling. Although 6-PP can also stimulate plant defense-related processes (Lazazzara et al., 2021), the present agar assay demonstrates only direct fungal growth suppression and does not establish that 6-PP accounted for the activity of the complete P1 secretome. Because targeted chemical profiling of the active P1 cultures and cell-free preparations was not performed, their active constituents remain unresolved. Future studies should combine GC–MS and LC–MS/MS profiling with bioactivity-guided fractionation, quantitative comparison with commercial 6-PP, and validation in cotton plants.

Antagonism toward M. phaseolina depended on the assay: R. arrhizus inhibited growth in the confrontation and volatile assays, whereas N. solani produced the strongest inhibition in the culture-conditioned medium assay. *Aspergillus* spp. are among the most chemically characterized fungi, producing a wide array of bioactive secondary metabolites (Liu et al., 2017). *A. niger* has a well-documented capacity to secrete organic acids and diverse secondary metabolites that can influence competing soil-borne fungi through pH modulation and metabolic interference, consistent with the strong antagonistic activity observed here (Schuster et al., 2002; Frisvad et al., 2018).

The greenhouse seedling experiment validated several interaction patterns observed *in vitro*. Because the experiment ended 33 days after sowing, the findings are limited to early root colonization. Full-season greenhouse and field trials are required to determine whether these interactions persist and influence late-season symptom development or yield. Consistent with the latent nature of CRD (Degani et al., 2023a), early aboveground growth parameters were largely unaffected across treatments. However, qPCR-based tracking revealed differences in pathogen colonization. These findings suggest that selected root-associated fungi may influence early pathogen establishment before visible symptoms develop. Further validation is required, but the observed interactions highlight the value of molecular diagnostics for early disease assessment (Degani et al., 2023a).

The interactions observed here parallel those reported between *M. phaseolina* and the maize pathogen *M. maydis*, which share ecological niches but differ in host specialization (Degani et al., 2020, 2022). Previous studies have shown that competitive interactions among phytopathogens can modulate disease expression and colonization success (Köhl et al., 2019; Degani et al., 2024a). Viewing *M. phaseolina* through the lens of *M. maydis* ecology reinforces the concept that CRD severity is shaped by microbial context rather than pathogen presence alone. This comparative framework supports the broader applicability of pathobiome-driven management strategies across crops affected by *M. phaseolina*.

Collectively, the findings suggest that cotton CRD is associated with variable and context-dependent interactions among selected root-associated fungi. Biological control agents such as *Trichoderma* remain promising tools, but their deployment should consider their potential effects on resident root-associated microorganisms. Equally, certain non-target fungi may suppress *M. phaseolina*, whereas others may favor its persistence under specific conditions. As demonstrated here, integrating culture-dependent profiling with functional interaction assays can provide a useful framework for developing sustainable, context-specific disease-management strategies that go beyond single-agent control.

## 5 Conclusions

This study supports the view that CRD in cotton is influenced by interactions among root-associated fungi rather than by *M. phaseolina* acting alone. Field sampling identified associations among sampling time, symptom status, management regime, and patterns in the culture-recovered fungal collection. By combining field-based isolation of culture-recovered cotton root fungi with functional interaction assays and controlled greenhouse testing, we suggest that specific co-occurring fungi can either suppress or enable pathogen colonization during early plant development, even when no visible symptoms are present. The concentration-dependent activity of commercial 6-pentyl-α-pyrone against *M. phaseolina* additionally identifies a chemically characterized *Trichoderma*-associated natural product for further mechanistic and *in planta* evaluation. Although the results do not establish community-wide restructuring or causality—questions that require sequencing-based community profiling and full-season trials—they indicate that microbial competition may be an important ecological driver of pathogen establishment and underscore the value of molecular tools for detecting latent disease processes. Future disease-management strategies should therefore move beyond targeting *M. phaseolina* alone and instead consider how interventions affect root-associated microorganisms. Priority should be given to validating these interactions across diverse field conditions, developing multispecies biocontrol consortia, and integrating culture-based functional assays with next-generation microbiome profiling. Microbiome-informed strategies should therefore be evaluated as a potential route toward more durable, reliable, and sustainable control of CRD in cotton.

## Data Availability

The original contributions presented in the study are publicly available. This data can be found here: NCBI GenBank, accession PX913211–PX913267, PX899697–PX899706.
