# Supplementary material for: Dynamics of culture-recovered cotton root-associated fungi under charcoal rot disease and preventive management regimes
Source: Front Fungal Biol. 2026 Oct 12;7:1928692. doi: 10.3389/ffunb.2026.1928692 (PMC13696409; doi:10.3389/ffunb.2026.1928692)
Supplement: Supplementary file 1 [file DataSheet1.docx]

Supplementary Material

# 1 Supplementary Tables

**Table S1.** GenBank Reference Strains Supporting the Phylogenetic Identification of Fungal Isolates in This Study. ^1^

| Genus | Strain Type | Strain Name | Species | Accession (ITS) |
| --- | --- | --- | --- | --- |
| Talaromyces | Type | CBS 631.66 | *T. pinophilus* | [NR_111691.1](https://www.ncbi.nlm.nih.gov/nucleotide/NR_111691.1?report=genbank&log$=nucltop&blast_rank=2&RID=JATDPU29014) |
|  | Type | CBS 388.87 | *T. marneffei* | [NR_103671.2](https://www.ncbi.nlm.nih.gov/nucleotide/NR_103671.2?report=genbank&log$=nucltop&blast_rank=1&RID=JAU1RAXM016) |
| Aspergillus | Type | NRRL 13137 | *A. nomiae* | [NR_121218.1](https://www.ncbi.nlm.nih.gov/nucleotide/NR_121218.1?report=genbank&log$=nucltop&blast_rank=7&RID=JAVY01H2016) |
|  | Type | DTO 493-H5 | *A. hubkae* | [NR_200571.1](https://www.ncbi.nlm.nih.gov/nucleotide/NR_200571.1?report=genbank&log$=nuclalign&blast_rank=81&RID=JAUDM102014) |
|  | Type | ATCC 16883 | *A. flavus* | [NR_111041.1](https://www.ncbi.nlm.nih.gov/nucleotide/NR_111041.1?report=genbank&log$=nucltop&blast_rank=7&RID=JAVZW2G8014) |
|  | Type | ATCC 16888 | *A. niger* | [NR_111348.1](https://www.ncbi.nlm.nih.gov/nuccore/NR_111348.1?report=fasta) |
| Penicillium | Type | NRRL 1841 | *P. citrinum* | [NR_121224.1](https://www.ncbi.nlm.nih.gov/nucleotide/NR_121224.1?report=genbank&log$=nucltop&blast_rank=44&RID=JAWGVEX2014) |
|  | Type | CBS 113149 | *P. araracuarense* | [NR_119814.1](https://www.ncbi.nlm.nih.gov/nucleotide/NR_119814.1?report=genbank&log$=nucltop&blast_rank=2&RID=JAWV80NJ014) |
| Chaetomium | Type | CBS 160.62 | *C. globosum* | [NR_144851.1](https://www.ncbi.nlm.nih.gov/nuccore/NR_144851.1) |
|  | Type | CBS 155.52 | *C. cochliodes* | [NR_151835.1](https://www.ncbi.nlm.nih.gov/nuccore/NR_151835.1) |
| Gibellulopsis | Type | CBS 120949 | *G. nigrescens* | [NR_149327.1](https://www.ncbi.nlm.nih.gov/nuccore/NR_149327.1) |
|  | Type | CBS 144923 | *G. simonii* | [NR_161152.1](https://www.ncbi.nlm.nih.gov/nuccore/NR_161152.1) |
| Gloeotinia | Reference | GS01 | *G. temulenta* | [DQ235697.1](https://www.ncbi.nlm.nih.gov/nuccore/DQ235697.1) |
| Trichoderma | Type | CBS 433.97 | *T. asperellum* | [NR_130668.1](https://www.ncbi.nlm.nih.gov/nuccore/NR_130668.1) |
|  | Type | CBS 226.95 | *T. harzianum* | [NG_062835.1](https://www.ncbi.nlm.nih.gov/nuccore/NG_062835.1) |
| Fusarium | Type | CBS 221.76 | *F. fujikuroi* | [NR_111889.1](https://www.ncbi.nlm.nih.gov/nuccore/NR_111889.1) |
|  | Type | CBS 140079 | *F. solani* | [NR_163531.1](https://www.ncbi.nlm.nih.gov/nuccore/NR_163531.1) |
| Rhizopus | Type | CBS 112.07 | *R. arrhizus* | [NR_103595.1](https://www.ncbi.nlm.nih.gov/nuccore/NR_103595.1) |
|  | Type | CBS112.07 | *R. oryzae* | [AB097334.1](https://www.ncbi.nlm.nih.gov/nuccore/AB097334.1) |
| Alternaria | Type | CBS 107.38 | *A. burnsii* | [NG_069257.1](https://www.ncbi.nlm.nih.gov/nuccore/NG_069257.1) |
|  | Reference | CBS 916.96 | *A. alternata* | [KF465761.1](https://www.ncbi.nlm.nih.gov/nuccore/KF465761.1) |

^1^ Type strains represent the official nomenclatural anchors for each genus and species according to the International Code of Nomenclature for algae, fungi, and plants (ICN). Reference strains listed beneath each type strain correspond to widely used phylogenetic representatives employed in recent high-impact fungal taxonomy, comparative genomics, and systematics studies. Accession numbers refer to ITS sequences in the NCBI GenBank.

**Supplementary Table S2.** BLASTN identification of the isolates from this study. ^1^

| **Isolate** | **Primer** | **Species** | **NCBI Accession** | **Query cover** | **Percent identical** | **Sampling date** | **Symptom status** | **Protective treatment** |
| --- | --- | --- | --- | --- | --- | --- | --- | --- |
| *Ed1* | *ITS4* | *Aspergillus tubingensis* | [PQ644070.1](https://www.ncbi.nlm.nih.gov/nucleotide/PQ644070.1?report=genbank&log$=nucltop&blast_rank=1&RID=EH41PBAV014) | 99% | 98.43% | Mid | Asymptomatic | - |
|  | *ITS5* | *Aspergillus tubingensis* | [KP278182.1](https://www.ncbi.nlm.nih.gov/nucleotide/KP278182.1?report=genbank&log$=nucltop&blast_rank=3&RID=EJB2P5T6014) | 99% | 94.12% |  |  |  |
| *Ed2* | ITS4 | *Trichoderma asperellum* (putative) | [MH627285.1](https://www.ncbi.nlm.nih.gov/nucleotide/MH627285.1?report=genbank&log$=nucltop&blast_rank=1&RID=EH4JUS57014) | 80% | 95.71% | Mid | Asymptomatic | Bio |
|  | ITS5 | *Trichoderma asperellum* | [LC535111.1](https://www.ncbi.nlm.nih.gov/nucleotide/LC535111.1?report=genbank&log$=nucltop&blast_rank=1&RID=EH4M410B015) | 96% | 94.59% |  |  |  |
| *Ed3* | ITS4 | *Rhizopus arrhizus* | [PP068157.1](https://www.ncbi.nlm.nih.gov/nucleotide/PP068157.1?report=genbank&log$=nucltop&blast_rank=1&RID=EJ9H3GXH015) | 99% | 97.91% | Mid | Asymptomatic | Bio |
|  | ITS5 | *Rhizopus arrhizus* | [DQ641282.1](https://www.ncbi.nlm.nih.gov/nucleotide/DQ641282.1?report=genbank&log$=nucltop&blast_rank=1&RID=EJ9J54XW014) | 99% | 97.23% |  |  |  |
| *Ed4* | ITS4 | *Rhizopus arrhizus* | [PP068158.1](https://www.ncbi.nlm.nih.gov/nucleotide/PP068158.1?report=genbank&log$=nucltop&blast_rank=1&RID=EJ9T4S7T014) | 99% | 97.91% | Mid | Asymptomatic | - |
|  | ITS5 | *Rhizopus arrhizus* | [MN006657.1](https://www.ncbi.nlm.nih.gov/nucleotide/MN006657.1?report=genbank&log$=nucltop&blast_rank=1&RID=EJ9UB6HH015) | 99% | 97.57% |  |  |  |
| *Ed5* | 338F | *Bacillus subtilis* | [OQ346200.1](https://www.ncbi.nlm.nih.gov/nucleotide/OQ346200.1?report=genbank&log$=nucltop&blast_rank=2&RID=EJABAY7V014) | 100% | 93.52% | Mid | Asymptomatic | - |
|  | 806R | *Bacillus subtilis* | [KU551191.1](https://www.ncbi.nlm.nih.gov/nucleotide/KU551191.1?report=genbank&log$=nucltop&blast_rank=1&RID=EJAC0PNW014) | 100% | 99.25% |  |  |  |
| *Ed6* | 338F | *Agrobacterium tumefaciens* | [KM252935.1](https://www.ncbi.nlm.nih.gov/nucleotide/KM252935.1?report=genbank&log$=nucltop&blast_rank=2&RID=EJAHXVYC014) | 100% | 95.34% | Mid | Asymptomatic | - |
|  | 806R | *Agrobacterium tumefaciens* | [KC488177.1](https://www.ncbi.nlm.nih.gov/nucleotide/KC488177.1?report=genbank&log$=nucltop&blast_rank=2&RID=EJAJPSRT014) | 97% | 96.33% |  |  |  |
| *Ed7* | 338F | *Bacillus licheniformis* | [PQ856513.1](https://www.ncbi.nlm.nih.gov/nucleotide/PQ856513.1?report=genbank&log$=nucltop&blast_rank=1&RID=EJATDK66014) | 100% | 95.01% | Mid | Asymptomatic | - |
|  | 806R | *Bacillus licheniformis* | [PQ780390.1](https://www.ncbi.nlm.nih.gov/nucleotide/PQ780390.1?report=genbank&log$=nucltop&blast_rank=2&RID=EJAUJ2XR014) | 98% | 92.84% |  |  |  |
| *Ed10A* | ITS4 | *Trichoderma asperellum* | [JQ040324.1](https://www.ncbi.nlm.nih.gov/nucleotide/JQ040324.1?report=genbank&log$=nucltop&blast_rank=1&RID=EMPR4293015) | 99% | 98.91% | Mid | Asymptomatic | Chem,  Integ |
|  | ITS5 | *Trichoderma asperellum* | [KY764822.1](https://www.ncbi.nlm.nih.gov/nucleotide/KY764822.1?report=genbank&log$=nucltop&blast_rank=1&RID=EMPRTAVH015) | 100% | 95.57% |  |  |  |
| *Ed10B* | ITS4 | *Trichoderma harzianum* | [KU896364.1](https://www.ncbi.nlm.nih.gov/nucleotide/KU896364.1?report=genbank&log$=nucltop&blast_rank=2&RID=EMR47EGC014) | 100% | 100.00% | End | Asymptomatic | Bio |
|  | ITS5 | *Trichoderma harzianum* | [MF150092.1](https://www.ncbi.nlm.nih.gov/nucleotide/MF150092.1?report=genbank&log$=nucltop&blast_rank=1&RID=EMR4WABH015) | 100% | 99.66% |  |  |  |
| *Ed11A* | ITS4 | *Aspergillus tubingensis* | [MT883446.1](https://www.ncbi.nlm.nih.gov/nucleotide/MT883446.1?report=genbank&log$=nucltop&blast_rank=2&RID=EMRE48CN014) | 100% | 99.46% | Mid | Asymptomatic | Bio |
|  | ITS5 | *Aspergillus tubingensis* | [MT043791.1](https://www.ncbi.nlm.nih.gov/nucleotide/MT043791.1?report=genbank&log$=nucltop&blast_rank=1&RID=EMREZGJU014) | 100% | 97.83% |  |  |  |
| *Ed11B* | ITS4 | *Aspergillus niger* | [PQ047139.1](https://www.ncbi.nlm.nih.gov/nucleotide/PQ047139.1?report=genbank&log$=nucltop&blast_rank=2&RID=EMRNRH9X014) | 100% | 99.47% | End | Asymptomatic | Chem,  Integ |
|  | ITS5 | *Aspergillus niger* | [KF305744.1](https://www.ncbi.nlm.nih.gov/nucleotide/KF305744.1?report=genbank&log$=nucltop&blast_rank=1&RID=EMRPEWFR014) | 100% | 96.13% |  |  |  |
| *Ed12A* | ITS4 | *Talaromyces annesophieae* | [NR_170732.1](https://www.ncbi.nlm.nih.gov/nucleotide/NR_170732.1?report=genbank&log$=nucltop&blast_rank=1&RID=EMRWPT3T014) | 99% | 98.91% | Mid | Asymptomatic | Integ |
|  | ITS5 | *Talaromyces annesophieae* | [NR_170732.1](https://www.ncbi.nlm.nih.gov/nucleotide/NR_170732.1?report=genbank&log$=nucltop&blast_rank=7&RID=EMRXDDV8014) | 100% | 98.36% |  |  |  |
| *Ed12B* | ITS4 | *Talaromyces verruculosus* (putative) | [MK367753.1](https://www.ncbi.nlm.nih.gov/nucleotide/MK367753.1?report=genbank&log$=nucltop&blast_rank=9&RID=EMSD87AM014) | 89% | 99.60% | End | Asymptomatic | Integ |
|  | ITS5 | *Talaromyces verruculosus* | [OP781641.1](https://www.ncbi.nlm.nih.gov/nucleotide/OP781641.1?report=genbank&log$=nucltop&blast_rank=1&RID=EMSE0RV1014) | 100% | 96.17% |  |  |  |
| *Ed13A* | ITS4 | *Rhizopus arrhizus* | [OQ305630.1](https://www.ncbi.nlm.nih.gov/nucleotide/OQ305630.1?report=genbank&log$=nucltop&blast_rank=4&RID=EMSUPBW4014) | 100% | 99.50% | Mid | Asymptomatic | Chem |
|  | ITS5 | *Rhizopus arrhizus* | [OP161823.1](https://www.ncbi.nlm.nih.gov/nucleotide/OP161823.1?report=genbank&log$=nucltop&blast_rank=1&RID=EMSW4M60015) | 100% | 97.69% |  |  |  |
| *Ed13B* | ITS4 | *Aspergillus flavus* | [MF442408.1](https://www.ncbi.nlm.nih.gov/nucleotide/MF442408.1?report=genbank&log$=nucltop&blast_rank=1&RID=EP8SHWRJ014) | 100% | 100.00% | End | Asymptomatic | Chem,  Bio |
|  | ITS5 | *Aspergillus flavus* | [MZ670446.1](https://www.ncbi.nlm.nih.gov/nucleotide/MZ670446.1?report=genbank&log$=nucltop&blast_rank=3&RID=EP8TY8M8014) | 97% | 99.46% |  |  |  |
| *Ed14A* | 338F | *Enterobacter ludwigii* | [PX022375.1](https://www.ncbi.nlm.nih.gov/nucleotide/PX022375.1?report=genbank&log$=nucltop&blast_rank=7&RID=ERGZ54PG015) | 98% | 99.76% | End | Asymptomatic | - |
|  | 806R | *Enterobacter ludwigii* | [PX022375.1](https://www.ncbi.nlm.nih.gov/nucleotide/PX022375.1?report=genbank&log$=nucltop&blast_rank=1&RID=ERH03YRB015) | 100% | 100.00% |  |  |  |
| *Ed15A* | 338F | *Bacillus subtilis* | [OP268482.1](https://www.ncbi.nlm.nih.gov/nucleotide/OP268482.1?report=genbank&log$=nucltop&blast_rank=1&RID=ERHAVJMB014) | 100% | 99.29% | End | Asymptomatic | - |
|  | 806R | *Bacillus subtilis* | [KP165034.1](https://www.ncbi.nlm.nih.gov/nucleotide/KP165034.1?report=genbank&log$=nucltop&blast_rank=1&RID=ERHBGK5N014) | 100% | 100.00% |  |  |  |
| *Ed16A* | 338F | *Bacillus amyloliquefaciens* | [MW540792.1](https://www.ncbi.nlm.nih.gov/nucleotide/MW540792.1?report=genbank&log$=nucltop&blast_rank=1&RID=ERHMMXBH015) | 100% | 100.00% | End | Asymptomatic | - |
|  | 806R | *Bacillus amyloliquefaciens* | [MG136818.1](https://www.ncbi.nlm.nih.gov/nucleotide/MG136818.1?report=genbank&log$=nucltop&blast_rank=12&RID=ERHNCGNN015) | 96% | 99.29% |  |  |  |
| *Ed17A* | ITS4 | *Penicillium citrinum* | [JX232276.1](https://www.ncbi.nlm.nih.gov/nucleotide/JX232276.1?report=genbank&log$=nucltop&blast_rank=1&RID=EPAYNA2W014) | 95% | 99.34% | Mid | Asymptomatic | Integ |
|  | ITS5 | *Penicillium citrinum* | [MZ410312.1](https://www.ncbi.nlm.nih.gov/nucleotide/MZ410312.1?report=genbank&log$=nucltop&blast_rank=2&RID=EPAZ9ABB014) | 99% | 98.64% |  |  |  |
| *Ed17B* ^2^ | ITS4 | *Penicillium citrinum* (putative) | [LC368457.1](https://www.ncbi.nlm.nih.gov/nucleotide/LC368457.1?report=genbank&log$=nucltop&blast_rank=1&RID=EPB7HWHH014) | 86% | 99.52% | End | Asymptomatic | Chem,  Integ |
|  | ITS5 | *Penicillium citrinum* (putative) | [MH858073.1](https://www.ncbi.nlm.nih.gov/nucleotide/MH858073.1?report=genbank&log$=nucltop&blast_rank=1&RID=EPB858HD014) | 70% | 98.62% |  |  |  |
| *Ed18A* | ITS4 | *Aspergillus calidoustus* | [OW986160.1](https://www.ncbi.nlm.nih.gov/nucleotide/OW986160.1?report=genbank&log$=nucltop&blast_rank=1&RID=EPBDKYS5014) | 99% | 99.07% | Mid | Asymptomatic | Chem |
|  | ITS5 | *Aspergillus calidoustus* | [PQ644076.1](https://www.ncbi.nlm.nih.gov/nucleotide/PQ644076.1?report=genbank&log$=nucltop&blast_rank=1&RID=EPBE7KYE014) | 100% | 97.11% |  |  |  |
| *Ed18B* | ITS4 | *Gloeotinia sp.* | [MK406111.1](https://www.ncbi.nlm.nih.gov/nucleotide/MK406111.1?report=genbank&log$=nucltop&blast_rank=6&RID=EPBMYESU015) | 100% | 99.82% | End | Asymptomatic | Chem |
|  | ITS5 | *Gloeotinia sp.* | [MK406111.1](https://www.ncbi.nlm.nih.gov/nucleotide/MK406111.1?report=genbank&log$=nucltop&blast_rank=4&RID=EPBNMP9F014) | 99% | 98.89% |  |  |  |
| *Ed19A* | ITS4 | *Aspergillus niger* (putative) | [MN243684.1](https://www.ncbi.nlm.nih.gov/nucleotide/MN243684.1?report=genbank&log$=nucltop&blast_rank=3&RID=EPBZG04N014) | 93% | 99.40% | Mid | Asymptomatic | Bio |
|  | ITS5 | *Aspergillus niger* | [MW411858.1](https://www.ncbi.nlm.nih.gov/nucleotide/MW411858.1?report=genbank&log$=nucltop&blast_rank=3&RID=EPC089NG014) | 99% | 96.13% |  |  |  |
| *Ed20A* | ITS4 | *Alternaria alternata* | [OP601340.1](https://www.ncbi.nlm.nih.gov/nucleotide/OP601340.1?report=genbank&log$=nucltop&blast_rank=1&RID=EPCBGJPE015) | 99% | 99.82% | End | Asymptomatic | Integ |
|  | ITS5 | *Alternaria alternata* | [PQ065965.1](https://www.ncbi.nlm.nih.gov/nucleotide/PQ065965.1?report=genbank&log$=nucltop&blast_rank=1&RID=EPCC6U5G014) | 100% | 98.72% |  |  |  |
| *Ed21A* | ITS4 | *Alternaria alternata* | [OP055935.1](https://www.ncbi.nlm.nih.gov/nucleotide/OP055935.1?report=genbank&log$=nucltop&blast_rank=1&RID=EPCJU0S0014) | 100% | 100.00% | End | Asymptomatic | Chem, Integ |
|  | ITS5 | *Alternaria alternata* | [OQ449453.1](https://www.ncbi.nlm.nih.gov/nucleotide/OQ449453.1?report=genbank&log$=nucltop&blast_rank=3&RID=EPCKM5AN014) | 100% | 98.86% |  |  |  |
| *Ed22A* | ITS4 | *Aspergillus flavus* | [MW084624.1](https://www.ncbi.nlm.nih.gov/nucleotide/MW084624.1?report=genbank&log$=nucltop&blast_rank=2&RID=EPCTNJN8014) | 100% | 99.46% | End | Asymptomatic | Chem |
|  | ITS5 | *Aspergillus flavus* | [MK271279.1](https://www.ncbi.nlm.nih.gov/nucleotide/MK271279.1?report=genbank&log$=nucltop&blast_rank=1&RID=EPD1YG4X014) | 100% | 99.65% |  |  |  |
| *Ed23* ^2^ | ITS4 | *Aspergillus flavus* (putative) | [MH865709.1](https://www.ncbi.nlm.nih.gov/nucleotide/MH865709.1?report=genbank&log$=nucltop&blast_rank=1&RID=EPDGP8FG014) | 86% | 98.48% | End | Asymptomatic | Chem |
|  | ITS5 | *Aspergillus flavus* (putative) | [PV291678.1](https://www.ncbi.nlm.nih.gov/nucleotide/PV291678.1?report=genbank&log$=nucltop&blast_rank=1&RID=EPDJDEWT014) | 93% | 96.04% |  |  |  |
| *Ed24* | ITS4 | *Rhizopus arrhizus* | [MN839779.1](https://www.ncbi.nlm.nih.gov/nucleotide/MN839779.1?report=genbank&log$=nucltop&blast_rank=1&RID=EPDSB4M1015) | 98% | 99.49% | End | Asymptomatic | Integ |
|  | ITS5 | *Rhizopus arrhizus* | [OP161825.1](https://www.ncbi.nlm.nih.gov/nucleotide/OP161825.1?report=genbank&log$=nucltop&blast_rank=1&RID=EPDSW9AY014) | 100% | 97.47% |  |  |  |
| *Ed25* ^2^ | 338F | *Acidovorax sp.* | [MT255158.1](https://www.ncbi.nlm.nih.gov/nucleotide/MT255158.1?report=genbank&log$=nucltop&blast_rank=1&RID=ERGF4EWK014) | 100% | 90.12% | End | Asymptomatic | - |
|  | 806R | *Pseudomonas indoloxydans* (putative) | [OR447760.1](https://www.ncbi.nlm.nih.gov/nucleotide/OR447760.1?report=genbank&log$=nucltop&blast_rank=1&RID=ERGFB0M0014) | 91% | 90.53% |  |  |  |
| *Ed27* | ITS4 | *Trichoderma asperellum* (putative) | [OM681182.1](https://www.ncbi.nlm.nih.gov/nucleotide/OM681182.1?report=genbank&log$=nucltop&blast_rank=1&RID=EPDZRN6W014) | 90% | 98.28% | End | Asymptomatic | Chem |
|  | ITS5 | *Trichoderma asperellum* | [MW074108.1](https://www.ncbi.nlm.nih.gov/nucleotide/MW074108.1?report=genbank&log$=nucltop&blast_rank=1&RID=EPE0D03R014) | 100% | 97.82% |  |  |  |
| *Ed29* | 338F | *Agrobacterium tumefaciens* | [OP518463.1](https://www.ncbi.nlm.nih.gov/nucleotide/OP518463.1?report=genbank&log$=nucltop&blast_rank=3&RID=EPESJEVN014) | 98% | 98.94% | End | Asymptomatic | - |
|  | 806R | *Agrobacterium tumefaciens* | [CP048464.1](https://www.ncbi.nlm.nih.gov/nucleotide/CP048464.1?report=genbank&log$=nucltop&blast_rank=5&RID=EPET85YR014) | 100% | 98.17% |  |  |  |
| *Ed30* | ITS4 | *Pyronema domesticum* | [MZ159659.1](https://www.ncbi.nlm.nih.gov/nucleotide/MZ159659.1?report=genbank&log$=nucltop&blast_rank=2&RID=8FZZBWDC014) | 99% | 98.58% | End | Asymptomatic | Bio |
|  | ITS5 | *Pyronema domesticum* | [PP923767.1](https://www.ncbi.nlm.nih.gov/nucleotide/PP923767.1?report=genbank&log$=nucltop&blast_rank=1&RID=8G0GH2FU016) | 72% | 86.83% |  |  |  |
| *Ed31* | ITS4 | *Aspergillus versicolor* | [MF476054.1](https://www.ncbi.nlm.nih.gov/nucleotide/MF476054.1?report=genbank&log$=nucltop&blast_rank=1&RID=EPFBV6YB014) | 99% | 98.87% | End | Asymptomatic | Integ |
|  | ITS5 | *Aspergillus versicolor* | [HQ285619.1](https://www.ncbi.nlm.nih.gov/nucleotide/HQ285619.1?report=genbank&log$=nucltop&blast_rank=3&RID=EPFD61G8014) | 100% | 94.56% |  |  |  |
| *Ed40A* ^2^ | 338F | *Pseudomonas reinekei* (putative) | [MW855897.1](https://www.ncbi.nlm.nih.gov/nucleotide/MW855897.1?report=genbank&log$=nucltop&blast_rank=2&RID=EPFP65EP014) | 94% | 92.82% | End | Symptomatic | - |
|  | 806R | *Pseudomonas sp.* | [OM679347.1](https://www.ncbi.nlm.nih.gov/nucleotide/OM679347.1?report=genbank&log$=nucltop&blast_rank=1&RID=EPFS8780015) | 100% | 97.85% |  |  |  |
| *Ed41A* | 338F | *Enterobacter cloacae* | [LS991346.1](https://www.ncbi.nlm.nih.gov/nucleotide/LS991346.1?report=genbank&log$=nucltop&blast_rank=3&RID=EPG9P039014) | 98% | 96.78% | End | Symptomatic | Bio |
|  | 806R | *Enterobacter cloacae* | [KT582295.1](https://www.ncbi.nlm.nih.gov/nucleotide/KT582295.1?report=genbank&log$=nucltop&blast_rank=5&RID=EPGACN2T014) | 100% | 98.25% |  |  |  |
| *Ed43A* | ITS4 | *Rhizopus arrhizus* | [OP692731.1](https://www.ncbi.nlm.nih.gov/nucleotide/OP692731.1?report=genbank&log$=nucltop&blast_rank=1&RID=EPGTZ4FR014) | 100% | 99.66% | End | Symptomatic | Integ |
|  | ITS5 | *Rhizopus arrhizus* | [OQ305630.1](https://www.ncbi.nlm.nih.gov/nucleotide/OQ305630.1?report=genbank&log$=nucltop&blast_rank=1&RID=EPGUKWZ8014) | 100% | 98.81% |  |  |  |
| *Ed44A* | ITS4 | *Rhizopus arrhizus* | [MN010559.1](https://www.ncbi.nlm.nih.gov/nucleotide/MN010559.1?report=genbank&log$=nucltop&blast_rank=1&RID=EPGZWT0B014) | 99% | 99.49% | End | Symptomatic | Bio |
|  | ITS5 | *Rhizopus arrhizus* | [MT603890.1](https://www.ncbi.nlm.nih.gov/nucleotide/MT603890.1?report=genbank&log$=nucltop&blast_rank=1&RID=EPH0FMMU014) | 100% | 99.62% |  |  |  |
| *Ed45A* | ITS4 | *Trichoderma asperellum* | [MN783078.1](https://www.ncbi.nlm.nih.gov/nucleotide/MN783078.1?report=genbank&log$=nucltop&blast_rank=1&RID=EPHA65P0014) | 97% | 97.03% | End | Symptomatic | Integ |
|  | ITS5 | *Trichoderma asperellum* | [PV739302.1](https://www.ncbi.nlm.nih.gov/nucleotide/PV739302.1?report=genbank&log$=nucltop&blast_rank=1&RID=EPHB9CS8014) | 100% | 100.00% |  |  |  |
| *Ed45B* | ITS4 | *Trichoderma asperellum* (putative) | [MT409888.1](https://www.ncbi.nlm.nih.gov/nucleotide/MT409888.1?report=genbank&log$=nucltop&blast_rank=1&RID=EPHR1Z8U014) | 88% | 98.05% | End | Symptomatic | - |
|  | ITS5 | *Trichoderma asperellum* (putative) | [HQ671189.1](https://www.ncbi.nlm.nih.gov/nucleotide/HQ671189.1?report=genbank&log$=nucltop&blast_rank=3&RID=EPHRKKMB014) | 88% | 99.11% |  |  |  |
| *Ed46A* | ITS4 | *Alternaria alternata* | [MN646267.1](https://www.ncbi.nlm.nih.gov/nucleotide/MN646267.1?report=genbank&log$=nucltop&blast_rank=4&RID=EPHWDZTN015) | 100% | 92.72% | End | Symptomatic | Bio |
|  | ITS5 | *Alternaria alternata* | [PQ065965.1](https://www.ncbi.nlm.nih.gov/nucleotide/PQ065965.1?report=genbank&log$=nucltop&blast_rank=2&RID=EPHX2M6X014) | 100% | 99.25% |  |  |  |
| *Ed47A* | ITS4 | *Alternaria alternata* | [OP601334.1](https://www.ncbi.nlm.nih.gov/nucleotide/OP601334.1?report=genbank&log$=nucltop&blast_rank=1&RID=EPJ6M7CA014) | 100% | 99.63% | End | Symptomatic | Integ |
|  | ITS5 | *Alternaria alternata* | [PQ065965.1](https://www.ncbi.nlm.nih.gov/nucleotide/PQ065965.1?report=genbank&log$=nucltop&blast_rank=1&RID=EPJ7GXZJ014) | 100% | 99.26% |  |  |  |
| *Ed48A* | ITS4 | *Alternaria alternata* | [OM980199.1](https://www.ncbi.nlm.nih.gov/nucleotide/OM980199.1?report=genbank&log$=nucltop&blast_rank=1&RID=EPJCTEN1015) | 100% | 99.45% | End | Symptomatic | Bio |
|  | ITS5 | *Alternaria alternata* | [JN689942.1](https://www.ncbi.nlm.nih.gov/nucleotide/JN689942.1?report=genbank&log$=nucltop&blast_rank=1&RID=EPJDR2E6015) | 100% | 100.00% |  |  |  |
| *Ed49A* | ITS4 | *Alternaria alternata* | [OL691143.1](https://www.ncbi.nlm.nih.gov/nucleotide/OL691143.1?report=genbank&log$=nucltop&blast_rank=1&RID=EPJM457U015) | 100% | 100.00% | End | Symptomatic | Bio |
|  | ITS5 | *Alternaria alternata* | [MW802703.1](https://www.ncbi.nlm.nih.gov/nucleotide/MW802703.1?report=genbank&log$=nucltop&blast_rank=1&RID=EPJMR72B014) | 100% | 99.44% |  |  |  |
| *Ed50A* | ITS4 | *Alternaria alternata* | [PP343089.1](https://www.ncbi.nlm.nih.gov/nucleotide/PP343089.1?report=genbank&log$=nucltop&blast_rank=1&RID=EPJSN1TY014) | 100% | 99.63% | End | Symptomatic | Bio |
|  | ITS5 | *Alternaria alternata* | [MW802703.1](https://www.ncbi.nlm.nih.gov/nucleotide/MW802703.1?report=genbank&log$=nucltop&blast_rank=1&RID=EPJTGDF4014) | 100% | 99.06% |  |  |  |
| *Ed51A* | ITS4 | *Alternaria alternata* | [OR351529.1](https://www.ncbi.nlm.nih.gov/nucleotide/OR351529.1?report=genbank&log$=nucltop&blast_rank=1&RID=EPJZG05W014) | 100% | 99.26% | End | Symptomatic | Bio |
|  | ITS5 | *Alternaria alternata* | [JQ769290.1](https://www.ncbi.nlm.nih.gov/nucleotide/JQ769290.1?report=genbank&log$=nucltop&blast_rank=1&RID=EPK04BN5014) | 100% | 99.62% |  |  |  |
| *Ed52A* | ITS4 | *Alternaria alternata* | [OP315751.1](https://www.ncbi.nlm.nih.gov/nucleotide/OP315751.1?report=genbank&log$=nucltop&blast_rank=1&RID=EPK5RM6S014) | 99% | 99.81% | End | Symptomatic | Bio |
|  | ITS5 | *Alternaria alternata* | [MW802703.1](https://www.ncbi.nlm.nih.gov/nucleotide/MW802703.1?report=genbank&log$=nucltop&blast_rank=2&RID=EPK6CHCH014) | 98% | 98.47% |  |  |  |
| *Ed53A* | ITS4 | *Fusarium oxysporum*  *F. nirenbergiae* (putative) | [PV600886.1](https://www.ncbi.nlm.nih.gov/nucleotide/PV600886.1?report=genbank&log$=nucltop&blast_rank=1&RID=EPKC9NF7014)  [JW 192006](https://www.ncbi.nlm.nih.gov/nuccore/MZ890524.1) | 90% | 99.05%  99.55% | End | Symptomatic | Bio |
|  | ITS5 | *Fusarium oxysporum*  *F. nirenbergiae* | [PX106381.1](https://www.ncbi.nlm.nih.gov/nucleotide/PX106381.1?report=genbank&log$=nucltop&blast_rank=1&RID=EPKCVMAU014)  [LC2804](https://www.ncbi.nlm.nih.gov/nuccore/MW016594.1) | 99% | 97.62%  96.25% |  |  |  |
| *Ed54A* | ITS4 | *Alternaria alternata* | [PP343089.1](https://www.ncbi.nlm.nih.gov/nucleotide/PP343089.1?report=genbank&log$=nucltop&blast_rank=1&RID=EPKJHYYV014) | 99% | 99.26% | End | Symptomatic | Integ |
|  | ITS5 | *Alternaria alternata* | [PQ158689.1](https://www.ncbi.nlm.nih.gov/nucleotide/PQ158689.1?report=genbank&log$=nucltop&blast_rank=1&RID=EPKKA0MX014) | 100% | 91.95% |  |  |  |
| *Ed56A* | ITS4 | *Aspergillus carlsbadensis* | [OL711775.1](https://www.ncbi.nlm.nih.gov/nucleotide/OL711775.1?report=genbank&log$=nucltop&blast_rank=1&RID=EPM5UDZB015) | 100% | 98.04% | End | Symptomatic | Chem |
|  | ITS5 | *Aspergillus carlsbadensis* | [LT883512.1](https://www.ncbi.nlm.nih.gov/nucleotide/LT883512.1?report=genbank&log$=nucltop&blast_rank=1&RID=EPM69FU8014) | 100% | 100.00% |  |  |  |
| *Ed57A* ^2^ | ITS4 | *Cladosporium sp.* | [PV596928.1](https://www.ncbi.nlm.nih.gov/nucleotide/PV596928.1?report=genbank&log$=nucltop&blast_rank=1&RID=EPMAZBES015) | 99% | 99.61% | End | Symptomatic | Chem,  Integ |
|  | ITS5 | *Cladosporium cladosporioides* (putative) | [MG664761.1](https://www.ncbi.nlm.nih.gov/nucleotide/MG664761.1?report=genbank&log$=nucltop&blast_rank=2&RID=EPMBPV7C014) | 85% | 97.65% |  |  |  |
| *Ed59A* | ITS4 | *Chaetomium cochliodes* | [MN534819.1](https://www.ncbi.nlm.nih.gov/nucleotide/MN534819.1?report=genbank&log$=nucltop&blast_rank=2&RID=EPMJU0G7014) | 100% | 99.63% | End | Symptomatic | Integ |
|  | ITS5 | *Chaetomium cochliodes* | [MN534819.1](https://www.ncbi.nlm.nih.gov/nucleotide/MN534819.1?report=genbank&log$=nucltop&blast_rank=5&RID=EPMKCW8C015) | 100% | 99.05% |  |  |  |
| *Ed60A* | ITS4 | *Botryotrichum murorum* | [MH860918.1](https://www.ncbi.nlm.nih.gov/nucleotide/MH860918.1?report=genbank&log$=nucltop&blast_rank=1&RID=EPMUTME6014) | 99% | 99.59% | End | Symptomatic | Chem |
|  | ITS5 | *Botryotrichum murorum* | [MH860918.1](https://www.ncbi.nlm.nih.gov/nucleotide/MH860918.1?report=genbank&log$=nucltop&blast_rank=1&RID=EPMVAWNV014) | 100% | 97.03% |  |  |  |
| *Ed61A* | ITS4 | *Chaetomium globosum* | [MN844798.1](https://www.ncbi.nlm.nih.gov/nucleotide/MN844798.1?report=genbank&log$=nucltop&blast_rank=5&RID=EPN0GFS2015) | 100% | 99.39% | End | Symptomatic | Chem |
|  | ITS5 | *Chaetomium globosum* | [OP714515.1](https://www.ncbi.nlm.nih.gov/nucleotide/OP714515.1?report=genbank&log$=nucltop&blast_rank=3&RID=EPN1BYF9014) | 100% | 99.80% |  |  |  |
| *Ed62A* | ITS4 | *Alternaria alternata* | [OP315751.1](https://www.ncbi.nlm.nih.gov/nucleotide/OP315751.1?report=genbank&log$=nucltop&blast_rank=1&RID=EPNH7JWD014) | 100% | 99.26% | End | Symptomatic | Integ |
|  | ITS5 | *Alternaria alternata* | [MN596827.1](https://www.ncbi.nlm.nih.gov/nucleotide/MN596827.1?report=genbank&log$=nucltop&blast_rank=3&RID=EPNJDR8C015) | 100% | 98.82% |  |  |  |
| *Ed63A* | ITS4 | *Alternaria alternata* | [PP343089.1](https://www.ncbi.nlm.nih.gov/nucleotide/PP343089.1?report=genbank&log$=nucltop&blast_rank=1&RID=EPNTM30J015) | 100% | 90.00% | End | Symptomatic | Chem |
|  | ITS5 | *Alternaria alternata* | [MW802703.1](https://www.ncbi.nlm.nih.gov/nucleotide/MW802703.1?report=genbank&log$=nucltop&blast_rank=7&RID=EPNU6X79015) | 100% | 97.34% |  |  |  |
| *Ed64A* | ITS4 | *Alternaria alternata* | [ON711964.1](https://www.ncbi.nlm.nih.gov/nucleotide/ON711964.1?report=genbank&log$=nucltop&blast_rank=1&RID=EPP2X0GK014) | 100% | 100.00% | End | Symptomatic | Chem |
|  | ITS5 | *Alternaria alternata* | [OR563627.1](https://www.ncbi.nlm.nih.gov/nucleotide/OR563627.1?report=genbank&log$=nucltop&blast_rank=1&RID=EPP3FJFH014) | 100% | 99.63% |  |  |  |
| *Ed67A* | ITS4 | *Gibellulopsis nigrescens* | [MK361141.1](https://www.ncbi.nlm.nih.gov/nucleotide/MK361141.1?report=genbank&log$=nucltop&blast_rank=1&RID=EPPASNCY014) | 99% | 99.43% | End-  season | Symptomatic | Integ |
|  | ITS5 | *Gibellulopsis nigrescens* | [MK361141.1](https://www.ncbi.nlm.nih.gov/nucleotide/MK361141.1?report=genbank&log$=nucltop&blast_rank=2&RID=EPPBGNCA014) | 100% | 99.41% |  |  |  |
| *Ed68A* | ITS4 | *Neocosmospora solani* | [MT529387.1](https://www.ncbi.nlm.nih.gov/nucleotide/MT529387.1?report=genbank&log$=nucltop&blast_rank=1&RID=EPPHU1JD014)  [CBS 208.29](https://www.ncbi.nlm.nih.gov/nuccore/MH855046.1) | 99% | 98.51%  98.23% | End-  season | Symptomatic | Chem |
|  | ITS5 | *Neocosmospora solani* | [PV242055.1](https://www.ncbi.nlm.nih.gov/nucleotide/PV242055.1?report=genbank&log$=nucltop&blast_rank=1&RID=EPPJGHJF014)  [NRRL 46598](https://www.ncbi.nlm.nih.gov/nuccore/GU170648.1) | 100% | 98.46%  99.58% |  |  |  |
| *Ed69A* | ITS4 | *Fusarium redolens*  *F. rhizicola* | [PP295257.1](https://www.ncbi.nlm.nih.gov/nucleotide/PP295257.1?report=genbank&log$=nucltop&blast_rank=1&RID=EPPTG3XG014)  [CBS 148872](https://www.fusarium.org/page/TaxonomyDisplay/499) | 99% | 100.00%  98.69% | End | Symptomatic | Chem |
|  | ITS5 | *Fusarium redolens*  *F. spartum* | [KP868529.1](https://www.ncbi.nlm.nih.gov/nucleotide/KP868529.1?report=genbank&log$=nucltop&blast_rank=1&RID=EPPUMBF9014)  [NRRL 66896](https://www.fusarium.org/page/TaxonomyDisplay/207) | 100% | 100.00%  99.81% |  |  |  |
| *Ed70A* | ITS4 | *Aspergillus terreus* | [OQ338186.1](https://www.ncbi.nlm.nih.gov/nucleotide/OQ338186.1?report=genbank&log$=nucltop&blast_rank=1&RID=EPR16669014) | 99% | 98.79% | End | Symptomatic | Integ |
|  | ITS5 | *Aspergillus terreus* | [PP070334.1](https://www.ncbi.nlm.nih.gov/nucleotide/PP070334.1?report=genbank&log$=nucltop&blast_rank=1&RID=EPR1U0WA015) | 99% | 94.92% |  |  |  |
| *Ed71A* ^2^ | ITS4 | *Chaetomium grande* | [MT529682.1](https://www.ncbi.nlm.nih.gov/nucleotide/MT529682.1?report=genbank&log$=nucltop&blast_rank=1&RID=EPRAYA6Y014) | 99% | 99.09% | End | Symptomatic | Integ |
|  | ITS5 | *Chaetomium globosum* | [MN844799.1](https://www.ncbi.nlm.nih.gov/nucleotide/MN844799.1?report=genbank&log$=nucltop&blast_rank=1&RID=EPRC2X0G015) | 100% | 96.82% |  |  |  |
| *Ed72A* | ITS4 | *Chaetomium cochliodes* | [MN534819.1](https://www.ncbi.nlm.nih.gov/nucleotide/MN534819.1?report=genbank&log$=nucltop&blast_rank=2&RID=EPRR7SMD014) | 99% | 99.81% | End | Symptomatic | Chem |
|  | ITS5 | *Chaetomium cochliodes* | [MN534819.1](https://www.ncbi.nlm.nih.gov/nucleotide/MN534819.1?report=genbank&log$=nucltop&blast_rank=6&RID=EPRRVMSH014) | 100% | 98.85% |  |  |  |
| *Ed73A* | ITS4 | *Aspergillus niger* | [HQ850370.1](https://www.ncbi.nlm.nih.gov/nucleotide/HQ850370.1?report=genbank&log$=nucltop&blast_rank=1&RID=EPS03P4B014) | 99% | 99.82% | End | Symptomatic | Integ |
|  | ITS5 | *Aspergillus niger* | [KU323591.1](https://www.ncbi.nlm.nih.gov/nucleotide/KU323591.1?report=genbank&log$=nucltop&blast_rank=13&RID=EPS0Y9WB015) | 97% | 99.64% |  |  |  |
| *Ed75A* | ITS4 | *Talaromyces verruculosus* | [HQ608025.1](https://www.ncbi.nlm.nih.gov/nucleotide/HQ608025.1?report=genbank&log$=nucltop&blast_rank=3&RID=EPS97TH7014) | 100% | 99.10% | End | Symptomatic | Chem, Integ |
|  | ITS5 | *Talaromyces verruculosus* | [OP781641.1](https://www.ncbi.nlm.nih.gov/nucleotide/OP781641.1?report=genbank&log$=nucltop&blast_rank=1&RID=EPS9UNUR014) | 100% | 97.43% |  |  |  |
| *Ed76A* | ITS4 | *Chaetomium globosum* | [MN844798.1](https://www.ncbi.nlm.nih.gov/nucleotide/MN844798.1?report=genbank&log$=nucltop&blast_rank=4&RID=EPSHZ0DH014) | 100% | 98.68% | End | Symptomatic | Integ |
|  | ITS5 | *Chaetomium globosum* | [MK183809.1](https://www.ncbi.nlm.nih.gov/nucleotide/MK183809.1?report=genbank&log$=nucltop&blast_rank=1&RID=EPSJR824015) | 100% | 99.81% |  |  |  |
| *Ed77A* | ITS4 | *Chaetomium globosum* | [MH151027.1](https://www.ncbi.nlm.nih.gov/nucleotide/MH151027.1?report=genbank&log$=nucltop&blast_rank=1&RID=EPSU88EH014) | 100% | 99.82% | End | Symptomatic | Chem |
|  | ITS5 | *Chaetomium globosum* | [OM333476.1](https://www.ncbi.nlm.nih.gov/nucleotide/OM333476.1?report=genbank&log$=nucltop&blast_rank=1&RID=EPSUSY3Y014) | 98% | 97.00% |  |  |  |
| *Ed78A* | ITS4 | *Neocosmospora solani* | [ON351016.1](https://www.ncbi.nlm.nih.gov/nucleotide/ON351016.1?report=genbank&log$=nucltop&blast_rank=1&RID=EPT0DWYU014)  [CBS 208.29](https://www.ncbi.nlm.nih.gov/nuccore/MH855046.1) | 99% | 99.81%  100.00% | End | Symptomatic | Chem |
|  | ITS5 | *Neocosmospora solani* | [PV242055.1](https://www.ncbi.nlm.nih.gov/nucleotide/PV242055.1?report=genbank&log$=nucltop&blast_rank=1&RID=EPT14J8V014)  [NRRL 44924](https://www.ncbi.nlm.nih.gov/nuccore/GU250660.1) | 100% | 99.43%  99.80% |  |  |  |

^1^ Cotton root-associated bacterial and fungal isolates from field plots representing different charcoal rot disease management treatments - azoxystrobin (Chem) and Trichoderma (Bio), in combination (Integ) or individually (Degani et al. 2024b). Asymptomatic and symptomatic plants were randomly selected. Molecular identification was based on ITS sequences for fungi and 16S rRNA gene sequences for bacteria, using the NCBI GenBank BLASTN search (National Center for Biotechnology Information, Bethesda, MD, USA, at: [http://www.ncbi.nlm.nih.gov](http://www.ncbi.nlm.nih.gov/), accessed on 15 January 2026). For Fusarium species identification, the Fusarioid-ID database pairwise alignment (available at [www.fusarium.org](http://www.fusarium.org), accessed 15 January, 2026) was utilized. **²** For isolates showing inconsistent ITS4/ITS5 BLAST results or insufficient sequence similarity, taxonomic identification is restricted to the genus level, unless species-level resolution is supported by clustering with ICN-recognized type or well-established reference strains.

**Supplementary** **Table S3**. Accession numbers of root-associated fungal and bacterial isolates recovered from cotton roots.

Fungi:

Isolate	ITS region NCBI accession numbers

Ed1	[PX913211](https://www.ncbi.nlm.nih.gov/nuccore/PX913211)

Ed2	[PX913212](https://www.ncbi.nlm.nih.gov/nuccore/PX913212)

Ed3	[PX913213](https://www.ncbi.nlm.nih.gov/nuccore/PX913213)

Ed4	[PX913214](https://www.ncbi.nlm.nih.gov/nuccore/PX913214)

Ed10A	[PX913215](https://www.ncbi.nlm.nih.gov/nuccore/PX913215)

Ed10B	[PX913216](https://www.ncbi.nlm.nih.gov/nuccore/PX913216)

Ed11A	[PX913217](https://www.ncbi.nlm.nih.gov/nuccore/PX913217)

Ed11B	[PX913218](https://www.ncbi.nlm.nih.gov/nuccore/PX913218)

Ed12A	[PX913219](https://www.ncbi.nlm.nih.gov/nuccore/PX913219)

Ed12B	[PX913220](https://www.ncbi.nlm.nih.gov/nuccore/PX913220)

Ed13A	[PX913221](https://www.ncbi.nlm.nih.gov/nuccore/PX913221)

Ed13B	[PX913222](https://www.ncbi.nlm.nih.gov/nuccore/PX913222)

Ed17A	[PX913223](https://www.ncbi.nlm.nih.gov/nuccore/PX913223)

Ed17B	[PX913224](https://www.ncbi.nlm.nih.gov/nuccore/PX913224)

Ed18A	[PX913225](https://www.ncbi.nlm.nih.gov/nuccore/PX913225)

Ed18B	[PX913226](https://www.ncbi.nlm.nih.gov/nuccore/PX913226)

Ed19A	[PX913227](https://www.ncbi.nlm.nih.gov/nuccore/PX913227)

Ed20A	[PX913228](https://www.ncbi.nlm.nih.gov/nuccore/PX913228)

Ed21A	[PX913229](https://www.ncbi.nlm.nih.gov/nuccore/PX913229)

Ed22A	[PX913230](https://www.ncbi.nlm.nih.gov/nuccore/PX913230)

Ed23	[PX913231](https://www.ncbi.nlm.nih.gov/nuccore/PX913231)

Ed24	[PX913232](https://www.ncbi.nlm.nih.gov/nuccore/PX913232)

Ed27	[PX913233](https://www.ncbi.nlm.nih.gov/nuccore/PX913233)

Ed30	[PX913234](https://www.ncbi.nlm.nih.gov/nuccore/PX913234)

Ed31	[PX913235](https://www.ncbi.nlm.nih.gov/nuccore/PX913235)

Ed43A	[PX913236](https://www.ncbi.nlm.nih.gov/nuccore/PX913236)

Ed44A	[PX913237](https://www.ncbi.nlm.nih.gov/nuccore/PX913237)

Ed45A	[PX913238](https://www.ncbi.nlm.nih.gov/nuccore/PX913238)

Ed45B	[PX913239](https://www.ncbi.nlm.nih.gov/nuccore/PX913239)

Ed46A	[PX913240](https://www.ncbi.nlm.nih.gov/nuccore/PX913240)

Ed47A	[PX913241](https://www.ncbi.nlm.nih.gov/nuccore/PX913241)

Ed48A	[PX913242](https://www.ncbi.nlm.nih.gov/nuccore/PX913242)

Ed49A	[PX913243](https://www.ncbi.nlm.nih.gov/nuccore/PX913243)

Ed50A	[PX913244](https://www.ncbi.nlm.nih.gov/nuccore/PX913244)

Ed51A	[PX913245](https://www.ncbi.nlm.nih.gov/nuccore/PX913245)

Ed52A	[PX913246](https://www.ncbi.nlm.nih.gov/nuccore/PX913246)

Ed53A	[PX913247](https://www.ncbi.nlm.nih.gov/nuccore/PX913247)

Ed54A	[PX913248](https://www.ncbi.nlm.nih.gov/nuccore/PX913248)

Ed56A	[PX913249](https://www.ncbi.nlm.nih.gov/nuccore/PX913249)

Ed57A	[PX913250](https://www.ncbi.nlm.nih.gov/nuccore/PX913250)

Ed59A	[PX913251](https://www.ncbi.nlm.nih.gov/nuccore/PX913251)

Ed60A	[PX913252](https://www.ncbi.nlm.nih.gov/nuccore/PX913252)

Ed61A	[PX913253](https://www.ncbi.nlm.nih.gov/nuccore/PX913253)

Ed62A	[PX913254](https://www.ncbi.nlm.nih.gov/nuccore/PX913254)

Ed63A	[PX913255](https://www.ncbi.nlm.nih.gov/nuccore/PX913255)

Ed64A	[PX913256](https://www.ncbi.nlm.nih.gov/nuccore/PX913256)

Ed67A	[PX913257](https://www.ncbi.nlm.nih.gov/nuccore/PX913257)

Ed68A	[PX913258](https://www.ncbi.nlm.nih.gov/nuccore/PX913258)

Ed69A	[PX913259](https://www.ncbi.nlm.nih.gov/nuccore/PX913259)

Ed70A	[PX913260](https://www.ncbi.nlm.nih.gov/nuccore/PX913260)

Ed71A	[PX913261](https://www.ncbi.nlm.nih.gov/nuccore/PX913261)

Ed72A	[PX913262](https://www.ncbi.nlm.nih.gov/nuccore/PX913262)

Ed73A	[PX913263](https://www.ncbi.nlm.nih.gov/nuccore/PX913263)

Ed75A	[PX913264](https://www.ncbi.nlm.nih.gov/nuccore/PX913264)

Ed76A	[PX913265](https://www.ncbi.nlm.nih.gov/nuccore/PX913265)

Ed77A	[PX913266](https://www.ncbi.nlm.nih.gov/nuccore/PX913266)

Ed78A	[PX913267](https://www.ncbi.nlm.nih.gov/nuccore/PX913267)

Bacteria:

Isolate	16S rRNA gene NCBI accession numbers

Ed5	[PX899697](https://www.ncbi.nlm.nih.gov/nuccore/PX899697)

Ed6	[PX899698](https://www.ncbi.nlm.nih.gov/nuccore/PX899698)

Ed7	[PX899699](https://www.ncbi.nlm.nih.gov/nuccore/PX899699)

Ed14A	[PX899700](https://www.ncbi.nlm.nih.gov/nuccore/PX899700)

Ed15A	[PX899701](https://www.ncbi.nlm.nih.gov/nuccore/PX899701)

Ed16A	[PX899702](https://www.ncbi.nlm.nih.gov/nuccore/PX899702)

Ed25	[PX899703](https://www.ncbi.nlm.nih.gov/nuccore/PX899703)

Ed29	[PX899704](https://www.ncbi.nlm.nih.gov/nuccore/PX899704)

Ed40A	[PX899705](https://www.ncbi.nlm.nih.gov/nuccore/PX899705)

Ed41A	[PX899706](https://www.ncbi.nlm.nih.gov/nuccore/PX899706)

**Supplementary** **Table S4**. Greenhouse seedling emergence and canopy development. ^1^

| **Measure** | **Non-inoculated** | ***M. phaseolina*-only** | *An* ***+***  ***Mp*** | *An* | *Aa* ***+***  ***Mp*** | *Aa* | *Ra* ***+***  ***Mp*** | *Ra* | *Ns* ***+***  ***Mp*** | *Ns* |
| --- | --- | --- | --- | --- | --- | --- | --- | --- | --- | --- |
| **Seedling emergence at day 6** | 5.0  ± 0.0 | 5.0  ± 0.0 | 4.9 ± 0.14 | 5.0  ± 0.0 | 4.9  ± 0.14 | 5.0  ± 0.0 | 4.9  ± 0.14 | 5.0  ± 0.0 | 5.0  ± 0.0 | 5.0  ± 0.0 |
| **Seedling emergence at day 13** | 5.0  ± 0.0 | 5.0  ± 0.0 | 5.0  ± 0.0 | 5.0  ± 0.0 | 5.0  ± 0.0 | 5.0  ± 0.0 | 4.9  ± 0.14 | 5.0  ± 0.0 | 5.0  ± 0.0 | 5.0  ± 0.0 |
| **Canopy cover day 13** | 8.7%  ± 1.3 | 9%  ± 1.4 | 12%  ± 1.2 | 9.3% ± 0.6 | 9.4% ± 0.7 | 12%  ± 1.5 | 11%  ± 1.1 | 8.7% ± 1.2 | 9.1% ± 1.2 | 9.4% ± 1.5 |
| **Canopy cover day 33** | 33%  ± 5.8 | 30%  ± 3.2 | 34%  ± 2.5 | 38%  ± 3.9 | 35%  ± 4.2 | 31%  ± 2.4 | 32%  ± 3.4 | 27%  ± 2.8 | 37%  ± 2.7 | 33%  ± 2.1 |

^1^ Cotton seedlings were inoculated with *Macrophomina phaseolina* (*Mp*; infected control) alone or together with *Aspergillus niger* (*An*), *Alternaria alternata* (*Aa*), *Rhizopus arrhizus* (*Ra*), or *Neocosmospora solani* (*Ns*). Non-inoculated plants served as controls. Seedling emergence was assessed at 6 and 13 days after sowing (DAS), and canopy cover was quantified at 13 and 33 DAS. Data are presented as mean ± SE, with seven independent pots per treatment at 6 and 13 DAS and 6–7 pots at 33 DAS. Emergence did not differ among treatments at 6 DAS (Kruskal–Wallis, *H*(9) = 7.209, *p* = 0.6154, ε² = 0.000) or 13 DAS (*H*(9) = 9.000, *p* = 0.4373, ε² = 0.000). No treatment differences were detected in canopy cover at 13 DAS (Kruskal–Wallis, *H*(9) = 7.695, *p* = 0.5651, ε² = 0.000) or 33 DAS (one-way ANOVA, *F*(9, 56) = 0.8940, *p* = 0.5366, η² = 0.1256). Because none of the omnibus tests were significant, post hoc comparisons were not performed.

**Supplementary Table S5.** Panel-level statistical reporting for the in vitro and greenhouse experiments

| **Figure / panel** | **Outcome / measurement** | **Assumption checks** | **Omnibus test** | **P value / status** | **Effect size** | **Post hoc test** |
| --- | --- | --- | --- | --- | --- | --- |
| Figure 7A | *M. phaseolina*, day 4 | Shapiro–Wilk: P = 0.1303; Brown–Forsythe: P = 0.0745 | One-way ANOVA: F(4, 18) = 47.91 | <0.0001 | η² = 0.9141 | Tukey |
| Figure 7B | *A. niger*, days 4–6 | Shapiro–Wilk: P = 0.5123; Brown–Forsythe: P = 0.5175 | One-way ANOVA: F(4, 20) = 13.98 | <0.0001 | η² = 0.7365 | Tukey |
| Figure 7C | *A. alternata*, days 4–6 | Shapiro–Wilk: P = 0.9892; Brown–Forsythe: P = 0.5283 | One-way ANOVA: F(4, 21) = 43.44 | <0.0001 | η² = 0.8922 | Tukey |
| Figure 7D | *R. arrhizus*, days 4–6 | Shapiro–Wilk: P = 0.2077; Brown–Forsythe: P = 0.0173 | Welch’s ANOVA: W(4, 9.500) = 243.8 | <0.0001 | η² = 0.9366 | Dunnett’s T3 multiple-comparisons test |
| Figure 7E | *N. solani*, days 4–6 | Shapiro–Wilk: P = 0.0415; Brown–Forsythe: P = 0.0260 | Kruskal–Wallis: H(4) = 22.62 | 0.0002 (approx.) | ε² = 0.8867 | Dunn |
| Figure 8A | *M. phaseolina*, day 2 | Shapiro–Wilk: P = 0.7154; Brown–Forsythe: P = 0.0876 | One-way ANOVA: F(4, 20) = 27.37 | <0.0001 | η² = 0.8456 | Tukey |
| Figure 8B | *A. niger*, day 2 | Shapiro–Wilk: P = 0.0144; Brown–Forsythe: P = 0.0189 | Kruskal–Wallis: H(4) = 17.94 | 0.0013 (approx.) | ε² = 0.7337 | Dunn |
| Figure 8C | *A. alternata*, day 2 | Shapiro–Wilk: P = 0.0854; Brown–Forsythe: P = 0.1167 | One-way ANOVA: F(4, 20) = 13.52 | <0.0001 | η² = 0.7300 | Tukey |
| Figure 8D | *R. arrhizus*, day 2 | Shapiro–Wilk: P = 0.0002; Brown–Forsythe: P = 0.2400 | Kruskal–Wallis: H(4) = 18.84 | 0.0008 (approx.) | ε² = 0.7420 | Dunn |
| Figure 8E | *N. solani*, day 2 | Shapiro–Wilk: P = 0.9437; Brown–Forsythe: P = 0.3944 | One-way ANOVA: F(4, 20) = 107.9 | <0.0001 | η² = 0.9557 | Tukey |
| Figure 9A | *M. phaseolina*, day 2 | Shapiro–Wilk: P = 0.3888; Brown–Forsythe: P = 0.3317 | One-way ANOVA: F(4, 20) = 22.41 | <0.0001 | η² = 0.8176 | Tukey |
| Figure 9B | *A. niger*, day 2 | Shapiro–Wilk: P = 0.0192; Brown–Forsythe: P = 0.4102 | Kruskal–Wallis: H(4) = 18.84 | 0.0008 (approx.) | ε² = 0.7420 | Dunn |
| Figure 9C | *A. alternata*, day 2 | Shapiro–Wilk: P = 0.0376; Brown–Forsythe: P = 0.7486 | Kruskal–Wallis: H(4) = 20.39 | 0.0004 (approx.) | ε² = 0.8195 | Dunn |
| Figure 9D | *R. arrhizus*, day 1 | Shapiro–Wilk: P = 0.1956; Brown–Forsythe: P = 0.6944 | One-way ANOVA: F(4, 20) = 1.220 | 0.3338 (ns) | η² = 0.1961 | Tukey |
| Figure 9E | *N. solani*, day 2 | Shapiro–Wilk: P = 0.4614; Brown–Forsythe: P = 0.8747 | One-way ANOVA: F(4, 20) = 42.65 | <0.0001 | η² = 0.8951 | Tukey |
| Figure 10A | *M. phaseolina* inhibition, day 3 | Shapiro–Wilk: P = 0.0044; Brown–Forsythe: P = 0.0038 | Kruskal–Wallis: H(3) = 15.01 | 0.0018 (approx.) | ε² = 0.5222 | Dunn |
| Figure 10C | 6-PP assay, *M. phaseolina,* net mycelial growth (mm) at 24, 48, and 72 h | Sphericity not assumed; Geisser–Greenhouse correction applied, ε = 0.8337 | Two-way RM ANOVA: concentration × time, F(5.002, 33.35) = 4322; concentration, F(3, 20) = 11796; time, F(1.667, 33.35) = 14689 | All effects:  *p* < 0.0001 | η² = 0.1285 (interaction); 0.7253 (concentration); 0.1456 (time) | Dunnett’s test comparing each concentration with its corresponding time-matched control; all adjusted P values were < 0.0001 |
| Figure 10C | 6-PP assay, *M. maydis,* net mycelial growth (mm) at 24, 48, and 72 h | Sphericity not assumed; Geisser–Greenhouse correction applied, ε = 0.7641; treated groups had zero variance | Two-way RM ANOVA: concentration × time, F(1.528,15.28) = 5360; concentration, F(1,10) = 23768; time, F(1.528,15.28) = 5360 | All effects:  *p* < 0.0001 | η² = 0.1654 (interaction); 0.6685 (concentration); 0.1654 (time) | Šídák’s test comparing 400 µg mL⁻¹ with its time-matched control; all adjusted P values were < 0.0001 |
| Figure 12A | Shoot weight, day 33 | Shapiro–Wilk: P = 0.4443; Brown–Forsythe: P = 0.6185 | One-way ANOVA: F(9, 56) = 0.9126 | 0.5211 (ns) | η² = 0.1279 | Tukey |
| Figure 12B | Shoot length, day 33 | Shapiro–Wilk: P = 0.5094; Brown–Forsythe: P = 0.5053 | One-way ANOVA: F(9, 56) = 1.054 | 0.4107 (ns) | η² = 0.1448 | Tukey |
| Figure 12C | Number of leaves, day 33 | Shapiro–Wilk: P < 0.0001; Brown–Forsythe: P = 0.1390 | Kruskal–Wallis: H(9) = 9.946 | 0.3549 (approx.; ns) | ε² = 0.0169 | Dunn |
| Figure 12D | Relative *M. phaseolina* DNA, qPCR, day 33 | Shapiro–Wilk: P < 0.0001; Brown–Forsythe: P = 0.1401 | Kruskal–Wallis: H(9) = 17.74 | 0.0384 (approx.) | ε² = 0.1561 | Dunn |
| Figure 12D related values | Raw COX Ct values | Shapiro–Wilk: W = 0.9088,  p = 0.0001 | Kruskal–Wallis: H(9) = 20.09 | 0.0173 | ε² = 0.1981 | Dunn |
| Table S4 | Seedling emergence at day 6 | Shapiro–Wilk: *W* = 0.4231,  *p* < 0.0001 | Kruskal–Wallis: H(9) = 7.209 | 0.6154 | ε² = 0.000 | Not performed |
| Table S4 | Seedling emergence at day 13 | Shapiro–Wilk: *W* = 0.2405,  *p* < 0.0001 | Kruskal–Wallis: H(9) = 9.000 | 0.4373 | ε² = 0.000 | Not performed |
| Table S4 | Canopy cover at day 13 | Shapiro–Wilk: W = 0.9607,  p = 0.0274 | Kruskal–Wallis: H(9) = 7.695 | 0.5651 | ε² = 0.000 | Not performed |
| Table S4 | Canopy cover at day 33 | Shapiro–Wilk: W = 0.9874, p = 0.7425; Brown–Forsythe: F(9,56) = 1.256, p = 0.2811 | One-way ANOVA: F(9,56) = 0.8940 | 0.5366 | η² = 0.1256 | Not performed |

**Notes.** Biological sample sizes are given in the corresponding figure captions or table notes. Residual normality and variance homogeneity were assessed by the Shapiro–Wilk and Brown–Forsythe tests, respectively. Tukey’s, Dunnett’s T3, or Dunn’s post hoc tests were used as appropriate; pairwise *p* values were multiplicity-adjusted, and differences are indicated by lowercase letters in the figures. Approximate *p*-values are identified. For Welch ANOVA, η² is a descriptive effect size based on conventional sums of squares; for Kruskal–Wallis, ε² = (H − k + 1)/(N − k). Unless otherwise specified, only omnibus P values are shown. η², eta-squared; ε², epsilon-squared.

# 2 Supplementary Figures

**Figure S1**. Phylogenetic analysis of the ITS5 region of fungal root isolates (listed in Supplementary Table S2). Sequence alignment and phylogenetic reconstruction were performed using SeaView software version 5.05 (<http://doua.prabi.fr/software/seaview>; accessed 10 January 2026). The phylogenetic tree was inferred using the PhyML maximum-likelihood algorithm (Guindon et al. 2010) under default parameters, with 1000 bootstrap replications to assess branch support. The tree is presented as an unrooted clustering tree and was used to assess the placement of isolates relative to reference and type strains. A consensus tree was generated from all calculated trees. Bootstrap values are shown at the major branches. The scale bar represents 0.5 nucleotide substitutions per site.

**Figure S2**. Plate confrontation assay illustrating antagonistic interactions among selected root-associated fungal species (Supplementary Table S2). Dual-culture assays were conducted on potato dextrose agar (PDA) to evaluate direct antagonistic interactions between selected cotton root-associated fungi. Control plates (highlighted in green) were inoculated with the same species on both sides of the Petri dish. In each confrontation assay, the focal species (highlighted) was inoculated on the right side of the plate and challenged by the opposing species on the left side. The tested fungi included *Macrophomina phaseolina*, *Aspergillus niger*, *Alternaria alternata*, *Neocosmospora solani*, and *Rhizopus arrhizus*. Plates were incubated at 28 ± 1 °C in the dark and photographed after 4–8 days, depending on species-specific growth rates.
